# ASC-G4, an algorithm to calculate advanced structural characteristics of G-quadruplexes

**DOI:** 10.1093/nar/gkad060

**Published:** 2023-02-16

**Authors:** Marc Farag, Cédric Messaoudi, Liliane Mouawad

**Affiliations:** Chemistry and Modeling for the Biology of Cancer, CNRS UMR9187, INSERM U1196, Institut Curie, PSL Research University, Université Paris-Saclay, CS 90030, 91401 Orsay Cedex, France; Multimodal Imaging Center, CNRS UMS2016, INSERM US43, Institut Curie, PSL Research University, Université Paris-Saclay, CS 90030, 91401 Orsay Cedex, France; Chemistry and Modeling for the Biology of Cancer, CNRS UMR9187, INSERM U1196, Institut Curie, PSL Research University, Université Paris-Saclay, CS 90030, 91401 Orsay Cedex, France

## Abstract

ASC-G4 is an algorithm for the calculation of the advanced structural characteristics of G-quadruplexes (G4). It allows the unambiguous determination of the intramolecular G4 topology, based on the oriented strand numbering. It also resolves the ambiguity in the determination of the guanine glycosidic configuration. With this algorithm, we showed that the use of the C3’ or C5’ atoms to calculate the groove width in G4 is more appropriate than the P atoms and that the groove width does not always reflect the space available within the groove. For the latter, the minimum groove width is more appropriate. The application of ASC-G4 to 207 G4 structures guided the choices made for the calculations. A website based on ASC-G4 (http://tiny.cc/ASC-G4) was created, where the user uploads his G4 structure and gets its topology, the types of its loops and their lengths, the presence of snapbacks and bulges, the distribution of guanines in the tetrads and strands, the glycosidic configuration of these guanines, their rise, the groove widths, the minimum groove widths, the tilt and twist angles, the backbone dihedral angles, etc. It also provides a large number of atom-atom and atom-plane distances that are relevant to evaluating the quality of the structure.

## INTRODUCTION

**Figure 1.**
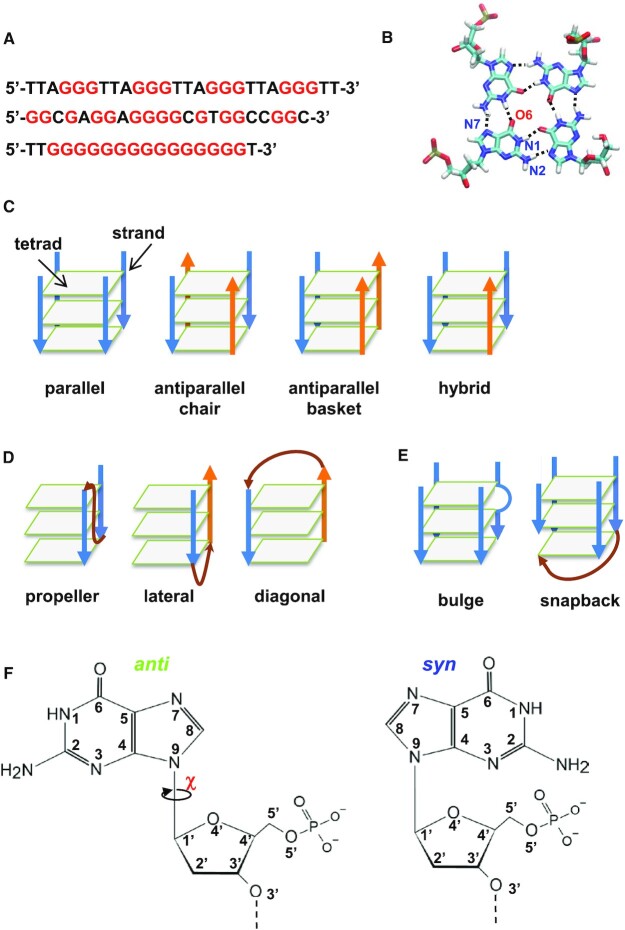
Structural features of G4. (**A**) Three G4 sequences, top, in the presence of four clear G-tracts, middle and bottom, in the absence of clear G-tracts. All guanines are in red. (**B**) A G4 tetrad, showing the Hoogsteen pairing between the four guanines. (**C**) Schematic representation of the different topology types of intramolecular G4s, where only the stems are shown; the loops are omitted for clarity. The stem is oriented from top to bottom. (**D**) Representation of the various loop types. (**E**) Representation of a bulge and a snapback. The tetrads are drawn as gray planes, the strands as arrows, blue for down and orange for up, and the loops are in brown. (**F**) 2D structures of *anti* and *syn* deoxyguanosines.

G-quadruplex (G4) is a secondary structure that takes place in a guanine-rich DNA or RNA nucleic acid sequence. G4 structures are polymorphic; they can be made of one or multiple strands (or chains) forming an intra- or intermolecular G-quadruplex, respectively. To form an intramolecular G4, the nucleic acid sequence should contain at least four G-tracts, which consist of successive guanines distributed in the sequence (Figure 1A, top). The interactions between guanine bases are of Hoogsteen type, consisting of a hydrogen-bond network in which atoms N1 and N2 of one guanine are the hydrogen-bond donors, whereas atoms O6 and N7 of the facing guanine are the hydrogen-bond acceptors (Figure 1B). In this Hoogsteen organization, four guanines form a tetrad (or quartet) (1). Generally, each of the four guanines of a G4 tetrad originates from a different G-tract (Figure 1A, top), and two or more tetrads stack on each other to constitute the G4 stem. In 3D structures, each G-tract usually forms a strand, but in some cases, either a strand is not made of all guanines of the G-tract, or it can be made of guanines from different G-tracts (Figure 1A, middle, and bottom). In intramolecular G4, the name ‘strand’ is given by analogy with the intermolecular G4; therefore, even an intramolecular G4 is made of four strands, although it consists of only one chain of nucleic acids. In the intermolecular G4, the strands are made of two (2,3) to four separate chains (4–6). In what follows, we focus on the intramolecular G4.

The classical and most spread classification of the G4 structures is the topology, based on the direction of the strands, this direction being up or down according to the numbering order of the successive guanines within the strand. Therefore, three main topologies are usually described: parallel, with all four strands in the same direction, antiparallel, with two strands in opposite directions, and hybrid, with one strand opposite to the three others (Figure 1C). The antiparallel topology has two forms, depending on the relative position of the opposite strands: when each strand is opposite to its two adjacent ones, it is called ‘chair’, and when two adjacent strands are in the same direction and opposite to the other two, it is called ‘basket’. The hybrid topology is also described to have two forms: form-1 and form-2, which differ in the position of the opposite strand (7). However, the difference between these two forms is not always clear and some structures were simply described to be either hybrid or (3 + 1) scaffold, without any specifications of their form. The strands are related by three different types of loops, propeller, lateral and diagonal (Figure 1D). The direction of a strand, up or down, is defined by the numbering order of its successive guanines. Usually, in a strand, successive guanines are numbered *n*, *n +*1, but in the presence of a bulge or a snapback (Figure 1E), the numbers of the two successive guanines in the strand are *n*, *n + m* + 1. In a bulge, *m* is the number of the additional nucleotides (nts) that constitute this bulge; it is usually equal to 1 or 2 nts, but it might be much more, as in the structure PDB ID: 7CLS (8), where *m* = 15. None of the bulge nts is involved in another strand. In a snapback, *m* is usually much >2, because, unlike in bulges, some of these additional nts constitute other strands. Most intramolecular G4s are made of a one-block stem, as the structures presented schematically in Figure 1C. However, in some cases, the G4 consists of two blocks, with two topologies, one for each block, as for the structure with the PDB ID: 2MS9 (9) (Figure S1 in the Supplementary Data).

It was shown that the G4 topology can be designed through the glycosidic configuration (gc) of the guanines (10). This configuration can be either *syn* or *anti*, depending on the orientation of the purine base relative to the sugar. In the *syn* configuration, the six-membered pyrimidine ring in the purine points toward or over the sugar ring, bringing close together atoms N3 and O5’, while in the *anti* configuration, it points away from it, taking these two atoms far from each other (11) (Figure 1F). This purine orientation is defined by the glycosidic bond angle (GBA), χ, around the glycosidic bond, C1’–N9. However, the literature concerning the relationship between the configuration and the ranges of the χ angle shows some confusion. According to the IUPAC-IUB definition (12) and to W. Saenger (11), for *syn* configuration, χ = 0 ± 90° and *anti*, χ = 180 ± 90°, whereas, in a quantum mechanics study (13), it was shown that the minimum energy for dG is around 60–80° for *syn* and 200–250° for *anti*. In addition, for the NMR resolution of the G4 structures, where the experimental signals are translated into restraints for the molecular dynamics (MD), in some cases, these restraints were centered on 60° and 240° for *syn*-G and *anti*-G (14,15), respectively, whereas in other cases they were centered on 0° and 180° (16), respectively. The ranges of the χ angle are discussed and clarified in the Results and Discussion section.

The *syn* and *anti* configurations of two adjacent guanines, i.e. two guanines from a Hoogsteen base-pair (Hbp), were described to determine the width of the groove between the two strands. However, usually, this width is not quantified and the atoms to be used for its calculation are not mentioned (17,18). This also is discussed in the Results and Discussion section.

X3dna-DSSR (19) (http://x3dna.org) is a website that was created to calculate nucleic acid structural parameters, like the local base-pair parameters, local step base-pair parameters, torsion angles, etc, but not the special characteristics of G4. A subdomain dedicated to G4, DSSR-G4DB (Dissecting the Spatial Structure of RNA – G4 Data Base) (http://g4.x3dna.org) emanated from this website. It is a database that gathers and calculates some specific structural information about published G4s, like the topology, the rise, the helical twist, etc, but not the groove widths or the presence of snapbacks. Since DSSR-G4DB is a database, the user cannot provide his own G4 structure, to obtain structural information. Hence the necessity of developing a website where the user uploads his G4 structure file to obtain all its important and specific structural characteristics (like the topology, the groove width, the tilt and twist angles, etc.). This can be very useful, not only for the analysis of published PDB structures but also for structures in refinement or obtained from MD simulations, to evaluate their quality. To our knowledge, there is no website dedicated to G4 to do such calculations in real-time. Therefore, we developed the algorithm ASC-G4 (advanced structural characteristics of G4) and deployed it as a user-friendly website at the following address: http://tiny.cc/ASC-G4.

Throughout its development, ASC-G4 was tested on 207 intramolecular structures taken from the PDB, to be sure that it works on different types of G4s. Many issues were found in some of these structures making the automatization more difficult than expected. This required the clarification and precise definition of each notion that is used. The software, the issues, and some definitions are explained in the Materials and Method section. The analysis of the G4 structures allowed the resolution of ambiguities concerning the topology, the snapbacks, and the glycosidic bond angle ranges. This is presented in the Results and Discussion section.

## MATERIALS AND METHODS

### Set of G4 structures

During the development of the program, we had to continuously test it to ensure that it worked properly. For this purpose, 207 intramolecular G4 structures, taken randomly from the Protein Data Bank (PDB) (http:/www.rscb.org/pdb/) (20), were considered. The sequence lengths of these G4s ranged between 12 and 84 nts. Some of them were redundant, but the structures were kept in the set, because it is well known that, in G4, the same sequence may adopt different structures and topologies, depending on experimental conditions (21–25).

### Program ASC-G4

The software package ASC-G4 was developed using AWK programming language. This package consisted of several parts: (i) identification of the tetrads, (ii) identification of the strands, (iii) ordering the tetrads, (iv) ordering the strands, (v) determination of gcs, (vi) identification of the G4 topology, (vii) calculation of the twist and tilt angles, (viii) calculation of the groove width, (ix) calculation of the minimum groove width, (x) calculation of the backbone and sugar dihedral angles and (xi) determination of the multimer types and characteristics. These parts are described below.

#### Identification of the tetrads

To identify the tetrads, the guanines that establish Hoogsteen-like interactions should be detected. For this purpose, the distance between hydrogen-bond donors (atoms N1 and N2) and hydrogen-bond acceptors (atoms O6 and N7) of all guanines were calculated (for atom names, refer to Figure 1). It was expected to only keep guanine pairs compatible with H-bond distances, with *d*_1_ and *d*_2_ <3.8 Å, where *d*_1_ is the N1–O6 distance and *d*_2_, the N2–N7 distance. However, in some structures, there were large Hoogsteen base-pair distances (up to almost 5.0 Å) resulting in very loose H-bonds, as in the structure PDB ID: 1OZ8 (26) (Supplementary Figure S2A). Therefore, considering the variability of these distances in the PDB structures, the limit of both distances, *d*_1_ and *d*_2_, was set to 5.0 Å although this is usually considered beyond the close contact between heavy atoms. Conversely, this limit was too loose for some other structures, where inadequate interactions were also detected between guanine pairs in adjacent strands, but in different tetrads. Therefore, the average of *d*_1_ and *d*_2_ distances, <*d*>, was also calculated, considering that the smallest average would correspond to the pair in the same tetrad. This was mostly the case, but not for all structures, as 2LPW (27) (Supplementary Figure S2B). To resolve this issue, another criterion was added, which is the belonging of the bases of the guanine pair to the same plane, or more precisely, the belonging of atom O6 to the plane of the facing pyrimidine ring of the purine. Therefore, for each guanine pair that fulfilled the distance criterion (*d*_1_ and *d*_2_ ≤ 5.0 Å), the plane of one guanine pyrimidine cycle was defined by three atoms, N1, O6, N7 and the distance of the facing guanine O6 atom to that plane, *d*_plane_, was calculated, to determine their co-planarity. The limit *d*_plane_ distance was set to 2.8 Å, under which the two bases were considered almost coplanar. Once more, this limit was loose but necessary to avoid missing some tetrads which are not fully planar, as in structure 148D (28) (Supplementary Figure S2C). However, in one structure, 6QJO (29), which is not part of our test set, the distance *d*_plane_ between a guanine and its Hoogsteen base pair was bigger than the distance *d*_plane_ between this guanine and a facing one located in a loop (Supplementary Figure S2D). Therefore, another criterion was added to discriminate between the guanines that belong to the same tetrad and the others, which is the distance between atoms O6. This distance should be <5 Å.

The output of this first step is a file gathering all the above information (*d*_1_, *d_2_*, < *d* >, *d*_O6–O6_ and *d*_plane_), in addition to the Hbp list, from which the distribution of guanines in the tetrads can be deduced, and the distances between the C3’ atoms of these guanosine pairs, as well as the distances between their C5’ atoms, and their P atoms. This file is shown in Supplementary Figure S3.

#### Identification of the strands

The second step consisted of the identification of the strands. This is also necessary for the ordering of the tetrads. So, to identify the guanines that belonged to the same strand, i.e. that stack over each other, the distances between the C1’ atoms (*d*_C1’–C1’_) of all guanosines that belonged to two distinct tetrads were calculated (Supplementary Figure S4A). This might be roughly considered representative of the distance between two successive tetrad planes because C1’ is in the base plane, and it does not rotate relative to the ribose, as does the base itself when adopting a *syn* or *anti* configuration. Once more, the limit distance, under which the two guanosines were considered to belong to the same strand, had to be loose enough to avoid missing any structures. This limit distance was equal to 7.5 Å. However, because of this loosening, in some cases, the distances between one guanosine of a tetrad and two guanosines of another tetrad, one from the same strand and one from another strand, were under this limit (dashed lines in Supplementary Figure S4A). For this reason, two other criteria were added for the identification of the stacking guanines within the stem: first, the distance between the centers of gravity (CG) of the purine heavy atoms, and second, the cosine of the angle between the purine planes (cosp). Several sets of thresholds that may be more or less restrictive on the distances or the planarity were considered. *d*_C1’–C1’_ < 7.5 Å, *d*_CG–CG_ < 6 Å and cosp < |0.87|, were sufficient for regular G4 structures, but for less parallel bases, the conditions were: *d*_C1’–C1’_ < 6 Å, *d*_CG–CG_ < 5 Å, and cosp < |0.82|. The absolute values of the cosines |0.87| and |0.82| correspond to about 30° and 35°, respectively. However, for other problematic structures, where the two ‘parallel’ guanine bases may be separated by an angle of up to 50.5° (cosp = 0.636 in 1MY9, for example, see Supplementary Figure S4B), the cosp condition was omitted, while keeping the most restrictive conditions on the distances. The choice of the thresholds is done automatically by the program by an iterative procedure.

Apart from the planarity, a supplementary difficulty in the determination of the stacking guanines was observed. In some cases, one guanine from a given strand stacks better over the guanine from another strand than over that from its own strand (Supplementary Figure S4C). Because of all the cited difficulties, several iterations between the strand identification and the tetrad identification were necessary to completely identify the stem composition.

A file listing the stacking nucleotides of the stem and summarizing the corresponding data is given (Supplementary Figure S5). It is not an exhaustive list of all stacking nucleotides of the chain, although it also comprises the results of some non-stem nucleotides that stack over or below the stem. If these nucleotides are C, T or U, the center of gravity is calculated for the pyrimidine base. Also, in this file, the rise of the stacking bases is given, the rise being the distance between the two successive base planes. But since sometimes the planes are not parallel, as described above, this distance is calculated between the CG of one base and the plane of the other base. The plane of a base is calculated from atoms N1, N3 and C5.

#### Ordering the tetrads

**Figure 2.**
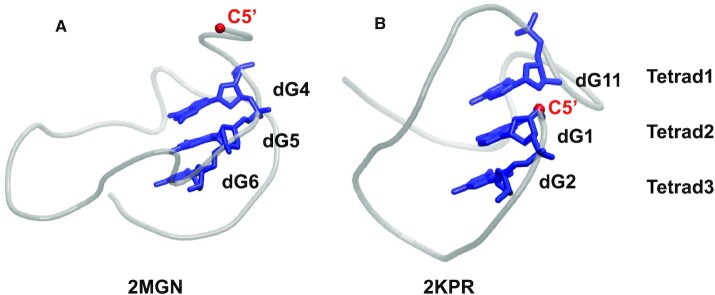
Ordering the tetrads. Two one-block structures, 2MGN (**A**) and 2KPR (**B**) are drawn as gray tubes, with their first strand as blue sticks. For each structure, the 5’ terminal is indicated by the C5’ atom of the first nucleotide, which is drawn as a red small sphere. As observed, in 2MGN (A), the first strand is continuous, whereas, in 2KPR (B), it is discontinuous. This is taken into account when ordering the tetrads.

To be able to order the tetrads, one should identify the first stem-guanine, i.e. the guanine that is at the junction of the first strand (that we call in this section the leading strand) and the first tetrad. Generally, this is the case for the one-block G4s and most of the two-block structures, but exceptionally, in the two-block G4s, if the nucleic chain starts in block 2, like in 1OZ8, the first stem-guanine is at the intersection of the first strand and the first tetrad of block 2, and therefore the second part of the strand becomes the leading one. In this study, the G4 stem is oriented from top to bottom, to always have the leading strand ‘down’, as shown in the schematic presentation of Figure 1C. In classical forms of G4 structures, the first (or top) tetrad is the 5’-tetrad, and therefore, the first strand consists of guanines with the lowest nt identifications among the stem guanines, running in the ascending order. This is illustrated by the structure 2MGN (30) (Figure 2A), where the first strand consists of dG4–dG5–dG6 (from top to bottom), and therefore the first stem-guanine is dG4. However, in some structures, the first tetrad is not the 5’-tetrad, as illustrated by 2KPR (14) (Figure 2B). In this structure, although dG1 is part of the stem, it does not belong to the first tetrad, but to the second one. In 2KPR, the first strand consists of dG11-dG1-dG2 (from top to bottom), and therefore, the first stem-guanine is dG11, which is not the lowest nt number. In all structures similar to 2KPR, dG1 is the second guanine of the first strand. So, identification of the first stem-guanine in such structures is based on stacking information. If dG1 is stacked between two nts, as it does in 2KPR (where dG1 is between dG11 and dG2), the first nt is the one among the two guanines with the highest identification (dG11 in 2KPR). However, by this means the first stem-guanine is still not identified for all types of intramolecular G4 structures. In some cases, although the 5’-terminal nt belongs to the top tetrad, it presents a discontinuity in the strand, corresponding to a snapback (see the example of 6XT7 in Figure 8D in the Results subsection entitled ‘Four different types of snapbacks’). In this case, the 5’-terminal nt is not the first stem-guanine, but the first stem-guanine is the next closest in the sequence to the 5’-terminal (dG4 in the example of 6XT7). The strand containing this guanine is therefore the leading strand (strand #1), and its direction is always down. Finally, the tetrads are ordered based on the knowledge of the first stem-guanine and the distribution of the guanines within the strands.

#### Ordering the strands

Now that the tetrads are ordered and the first strand identified, the other three strands should be numbered. As presented above, the G4 is always looked at from the top tetrad of the leading strand, down. In this orientation, strand number 2 can be located either to the right (anticlockwise rotation) or to the left (clockwise rotation) of strand 1. Please note that here, the clockwise rotation corresponds to the anticlockwise rotation presented by da Silva (18) since our G4 orientation is opposite to what is described in that article. Usually, the strands are not numbered, but here, to remove the topology ambiguities, we need to attribute a unique number to each strand, always in the same direction and without visualization of the structure. For the purpose of a clear explanation, we will consider the case of the right-handed helices, and adopt the clockwise rotation.

**Figure 3.**
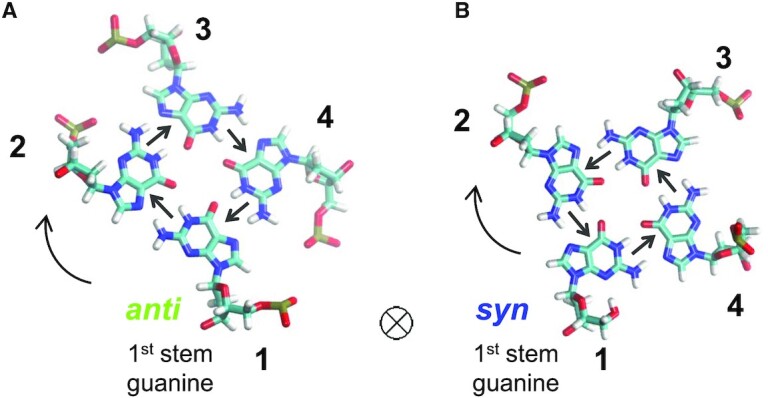
The orientation of the strands. Top view of two first tetrads, one starting with an *anti*-G (**A**), and one with a *syn*-G (**B**). The small straight arrows indicate the orientation of the H-bond donors in the Hoogsteen pairing. The big curved arrows indicate the orientation of the strand numbering. The strand numbers are in bold. The atoms’ color code is the following: C (cyan), N (blue), O (red), P (tan) and H (white).

To decide automatically which is the clockwise orientation, without visualization of the structure, an objective criterion is needed. This criterion is the gc of the first stem-guanine. Indeed, when looking at the backbone of the first guanine along its 5’ → 3’ direction, if its configuration is *anti*, by definition, to avoid the sugar, its hydrogen-bond donors (atoms N1 and N2) are oriented to the left,—as well as those of all guanines of the first tetrad—(Figure 3A), and if it is *syn* all H-bond donors of this tetrad are oriented to the right (Figure 3B). Based on this observation, the strands are ordered according to the first stem-guanine: if its configuration is *anti*, then strand 2 is the one toward which it orients its H-bond *donors*, and if it is *syn*, strand 2 is the one toward which it orients its H-bond *acceptors*. The numbering of strands 3 and 4 results from that of strand 2. This amounts to numbering the strands of most G4s in the clockwise orientation when looking at the stem from top to bottom. Despite this description that uses the G4 orientation in space, the order of the strands is actually based on an intrinsic characteristic of the G4, that is the gc of its first stem-guanine. Therefore, this strand numbering is independent of the G4 orientation in space.

#### Determination of the glycosidic configuration

**Figure 4.**
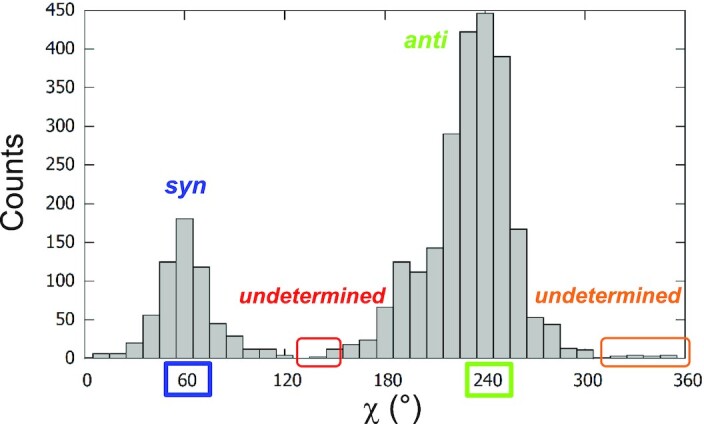
Histogram of the χ angles of the stem guanines from the 207 G4 structures, with a bin size of 10°. As observed, the bimodal distribution has two peaks at 60° for *syn*-G and 240° for *anti*-G. Between these two populations, there are much less populated regions that we qualified as undetermined.

To objectively order the strands in the same direction, it is therefore important to determine the gc. However, this is not obvious considering the inconsistency in the literature of the definition of the configuration based on the ranges of the χ angle, as presented in the Introduction. We, consequently, calculated the distribution of the χ angle in our set of 207 structures (Figure 4). The two peaks were not centered on 0° and 180°, for *syn* and *anti*, as expected from the IUPAC-IUB definition (12), but on 60° and 240°, respectively. Based on this distribution, the following ranges were used: the configuration was considered *syn* when 0° < χ ≤ 140°, (we prefer the equivalent but more concise mathematical notation ${\rm{\chi }} \in {] {0^\circ ,140^\circ } ]} )$ and *anti* when ${\rm{\chi }} \in {] {152^\circ ,300^\circ } ]}$. A short reminder about the bracket notation: the closed bracket indicates that the range includes the value, while the open bracket indicates that the value is excluded.

Between these two ranges, there are sparsely populated regions that we qualified as undetermined, ${\rm{\chi }} \in {] {140^\circ ,152^\circ } ]}$ or ${\rm{\chi }} \in {] {300^\circ ,360^\circ } ]}$. The χ angle is defined by the four atoms O4’–C1’–N9–C4. However, the configuration in the indeterminacy intervals was further refined as presented below.

In some G4 structures, the χ angle was not correct enough to determine the orientation of the base because of the erroneous position of the C1’ atom, which is one of the two central atoms of this torsional angle. As mentioned before, C1’ should be in the plane of the guanine base, but this is not always the case. To verify it, the distance between atom C1’ and the plane of the imidazole ring of the base was calculated. This plane was represented by the closest atoms of the base to C1’, i.e. N9-C4–C8 (for the atom names, refer to Figure 1F). For a distance bigger than 0.15 Å, C1’ was considered to be out of the base plane because this started to be visually observable (Supplementary Figure S6). In this case, the value of the χ angle was distorted. When the calculated χ is in the middle of the *syn* or *anti* ranges, the error does not modify the configuration type of the guanine, but when χ is on the edges, i.e. in the indeterminacy intervals, this is of importance and should be considered.

When χ is in the indeterminacy intervals, and it is not distorted (C1’ is in the base plane), two distances were used to finally provide the configuration, the distance between atoms N3 and O5’, *d*_N3–O5’_, and the distance between atoms H1’ and H8, *d*_H1’–H8_. The first distance generally reflects the fact that the base faces the sugar in the *syn* configuration and points away from it in the *anti* configuration. The second distance reflects the presence, for NMR structures, of a high-intensity NOESY cross-peak between atoms H1’ and H8 in the *syn* configuration, and the absence of such a peak in the *anti* configuration. Based on the calculations made on our set of 207 G4s, the limit distances, under which the configuration was considered *syn* and over which it was considered *anti*, were 5.5 Å for *d*_N3–O5’,_ and 3.2 Å for *d*_H1’–H8_. For most guanines located in the indeterminacy intervals that had a non-distorted χ angle, these two distances were near their respective limits and they pointed to contradictory configurations. Therefore, to decide which distance to use, their relative remotenesses from the limits were calculated, ${r}_{{\rm{N3 - O5}}^{\prime}} = {{( {{d}_{{\rm{N3 - O5}}^{\prime}} - 5.5} )} \mathord{/ {\vphantom {{( {{d}_{{\rm{N3 - O5}}^{\prime}} - 5.5} )} {5.5}}} \kern-\nulldelimiterspace} {5.5}}$ and ${r}_{{\rm{H1^{\prime} - H8}}} = {{( {{d}_{{\rm{H1^{\prime} - H8}}} - 3.2} )} \mathord{/ {\vphantom {{( {{d}_{{\rm{H1^{\prime} - H8}}} - 3.2} )} {3.2}}} \kern-\nulldelimiterspace} {3.2}}$, and the farthest was favored. So, if the absolute values of the remotenesses were $| {{r}_{{\rm{N3 - O5}}^{\prime}}} | >| {{r}_{{\rm{H1^{\prime} - H8}}}} |$, the final retained configuration was the one based on *d*_N3-O5’_, and if $| {{r}_{{\rm{H1^{\prime} - H8}}}} | >| {{r}_{{\rm{N3 - O5}}^{\prime}}} |$it was the one based on *d*_H1’–H8_.

When χ is in the indeterminacy intervals and it is distorted (C1’ is out of the base plane), the distance *d*_H1’–H8_ is not reliable as well, since H1’ is bound to C1’; *d*_N3-O5’_ is not reliable either because of some observed discrepancies with the χ angle, even when the latter is correct (see Results and Discussion). Therefore, when χ is in the indeterminacy intervals and it is distorted, the program gives the configuration as undetermined. However, for the first stem-guanine, the configuration cannot remain undetermined, since the *syn* or *anti* property defines the numbering of the strands. So, only in this case, if the configuration is undetermined, the distance *d*_H1’-H8_ is considered as the criterion to determine the configuration and therefore the strand numbering. When hydrogen atoms are missing like in crystal structures, *d*_H1’-H8_ is given as not available (NA), and in this case, for the first stem-guanine, the configuration is determined by *d*_N3–O5’_. This is only used for the orientation of the strands, but in the output files, the configuration is still given as undetermined.

To summarize, the resulting configuration is *syn* for ${\rm{\chi }} \in {] {0^\circ ,140^\circ } ]}$ and *anti* for ${\rm{\chi }} \in {] {152^\circ ,300^\circ } ]}$. But for ${\rm{\chi }} \in {] {140^\circ ,152^\circ } ]}$ or ${\rm{\chi }} \in {] {300^\circ ,360^\circ } ]}$, there are two distinct situations: if the distance between C1’ and the imidazole-ring plane is less than 0.15 Å, the configuration is the one obtained by either *d*_N3-O5’_ or *d*_H1’-H8_ after comparing the absolute value of their relative remoteness from their respective limit distances $| {{r}_{{\rm{N3 - O5}}^{\prime}}} |$, $| {{r}_{{\rm{H1^{\prime} - H8}}}} |$, but if C1’ is out of the imidazole plane, the configuration remains undetermined.

An output file is provided at this point (Supplementary Figure S7), which gathers the following information: the χ angle, the distances *d*_N3–O5’_ and *d*_H1’–H8_, followed by their respective predicted configurations, the distance of C1’ to the imidazole plane of the purine, and finally, the resulting configuration.

#### Identification of the G4 topology

As explained before, in ASC-G4, the structure is always looked at to have the direction of the first strand down (d), i.e. with an increasing nt number from top to bottom. For the other strands, their direction is based on the order of their guanine numbering, if it increases from top to bottom, the direction is down (d), and if it decreases, the direction is up (u). For structures like 2KPR (Figure 2B), even though the first stem-guanine number (dG11) is bigger than the following ones in the strand (dG1 and dG2), the direction of the strand is still considered down because two-thirds of the strand are oriented down.

Based on the up and down directions of the strands, the topology of the G4 is identified. Since the first strand is down by definition, all the possible combinations of the three other strands result in eight topologies: dddd = parallel, dudu = antiparallel-chair, duud = antiparallel-basket, dduu = antiparallel-basket2, ddud = hybrid1, dddu = hybrid2, dudd = hybrid3, and duuu = hybrid4. Since in ASC-G4 the ordering of the strands is unambiguous, these topologies are clearly distinguishable.

These topologies are defined for the one-block G4 structures. However, some structures consist of two blocks, which are observed when two successive tetrads present discontinuities (mostly different from bulges and snapbacks) in at least three strands. By definition, block 1 always starts with tetrad 1, and block 2 ends with the last tetrad. In the two-block structures, the strand directions might differ in the first block from those in the second block, and therefore, one such G4 has two topologies, one for each block. Since the first strand in these structures is made of two segments, the down orientation is based on the segment with the smallest nt identifications and, as mentioned above, the first stem-guanine is at the junction of the first tetrad in this block and the first strand. The identification of the first stem-guanine is provided in a separate downloadable file. Considering that the first stem-guanine might be located in either the first or the second block, and the direction of the first strand in this block is down, the direction of the first strand in the other block of the G4 can be either down or up. In the case it is up, all topology definitions are the same with reversed strand directions, since the first strand is up instead of down, as for structure 2MS9, for instance (Supplementary Figure S1). However, these structures may contain up to two snapbacks, and since each block is generally (but not always) made of two tetrads, the direction of the strand that contains the snapback is not obvious. Therefore, the direction of this strand is determined unambiguously based on the gc pattern of the tetrad that does not contain the snapback. For example, in 7D5E (31) (Supplementary Figure S8), in the second block, strands number 1, 2 and 3 are up, while strand 4 is made of dG15 and dG26. Because dG26 is a snapback, the direction of this strand is not obvious, it can be either up or down. Therefore, since the gcs of all the nucleotides of this block (excluding dG26, which is not considered) are *anti*, and it is known that, when all guanines in a structure are *anti* (excluding the snapback, which can be *syn* or *anti*), the structure is parallel, then the topology of this block is parallel. Consequently, the direction of this strand is up since the direction of the three other strands is up.

The loops that connect the strands follow the nt sequence. They are defined in a more general way than the usual propeller connecting two parallel strands, lateral, two adjacent antiparallel strands, diagonal, two opposite antiparallel strands, and the V-shaped loop, a special case of a propeller loop consisting of 0 nt. Here, the propeller loop connects two nts belonging to two adjacent strands and two different tetrads (whatever the length of the loop), the lateral loop connects two nts belonging to two adjacent strands and the same tetrad, and the diagonal loop connects two nts belonging to two non-adjacent strands and the same tetrad. This general definition allows overcoming some ambiguities when there are discontinuities in the strands. For instance, in 2KPR (see the Results), the loop that connects two adjacent strands (dG11 in strand 1 and dG12 in strand 4) with two opposite directions (strand 1 is down and strand 4 is up) is not a lateral loop, as would be expected, but a propeller loop. Therefore, with the general definition of the loops, this ambiguity is removed. In addition, information about the position of the lateral and diagonal loops, on the top (t) or at the bottom (b) of the stem, and about the direction of the propeller loops, from bottom to top, or from top to bottom is also given, as well as the progression of the loops, as a ‘+’ sign for the clockwise progression and a ‘–’ sign for the anticlockwise progression. Please note that the loop's position and progression are dependent on the orientation in space of the G4. Whereas ‘t’ and ‘b’ refer to the position in a top-to-bottom oriented G4, to be coherent with the output tables of ASC-G4, ‘+’ and ‘–’ refer to the progression in a bottom-to-top orientated G4, to be coherent with the previous publications that use the loop-based classification method, which was proposed by da Silva (18). ASC-G4 also identifies the bulges and snapbacks for all structures, and the linkers for the two-block structures. We define the linker as the shortest segment that connects the two parts of a strand, one from each block, without passing by any other stem guanines. Considering this definition, each strand might have, or not, a linker between the two blocks. The difference between a linker and a loop is that a linker connects two guanines from different blocks in the same strand, whereas a loop connects two guanines from different strands, whatever the blocks.

#### Calculation of the twist and tilt angles

In G4, the helix twist is the rotation of a tetrad relative to its successive one. To measure the twist angle, the most spread method is that described by Lu and Olson (2003) (32) and Reshetnikov et al. (2010) (33). In this method, the angle is calculated from the dot product between two C1’–C1’ vectors from two successive tetrads, *i* and *i* + 1, the C1’ atoms of each vector belonging to two adjacent guanosines of a Hbp. The issue with this method is that it does not allow access to the sign of the angle, which defines the direction of the G4 helix, viz. right-handed or left-handed.

To calculate this sign, a more general definition of the twist angle is used here, that is the angle of rotation of the bases around the helix axis. We call it the torsional twist angle to distinguish it from the usual twist angle. We adopted this definition, after reassuring that both calculations give similar results. The torsional twist angle is defined as the pseudo-dihedral angle between the bases around the G4 axis. So, for each strand in two successive tetrads (*i* and *i +*1), the pseudo-dihedral angle is C1’(*i*)-CG(*i*)-CG(*i* + 1)-C1’(*i* + 1), where C1’(*i*) and C1’(*i* + 1) are the C1’ atoms of two successive guanosines in a strand, and CG(*i*) and CG(*i* + 1) are the centers of gravity (CG) of the four O6 atoms in tetrads *i* and *i* + 1, respectively (Supplementary Figure S9A). The line relating CG(*i*) and CG(*i* + 1) corresponds to the local G4 axis. For each strand, the average (avg) and standard deviation (SD) are given. In addition, the avg and SD of the twist angles of all strands along a tetrad relative to the successive one are also given. When the latter avg is positive, the torsion is right-handed, otherwise, it is left-handed. Most structures have all their tetrads with the same twist orientation and therefore, the helix is either right-handed or left-handed. However, for some structures, like 2LA5 (34), there is a mix between left-handed and right-handed torsions, in which case the helix is given as a hybrid right-handed/left-handed helix.

The tilt angle, which is the angle between the tetrads and the strands is also calculated. It represents the inclination of the G4 quadri-helix relative to the tetrad planes. Therefore, the tilt angle is between two vectors, ${\vec{V}}_i$ and ${\vec{U}}_{i,i + 1}$, where ${\vec{V}}_i$ relates the C1’ atoms of two adjacent guanosines in tetrad *i*, and ${\vec{U}}_{i,i + 1}$ relates the C1’ atoms of two successive guanosines in the strand, belonging to tetrads *i* and *i* + 1. ${\vec{V}}_i$ and ${\vec{U}}_{i,i + 1}$ share the same C1’ atom as shown in Supplementary Figure S9B. This calculation is done for all four strands, and, for each strand and each tetrad, the average tilt angle and standard deviation are given.

#### Calculation of the groove width

By analogy with the dsDNA structures (35), the groove width in G4 is sometimes defined by the distance between the phosphorus atoms, P, at the 5’-end of two facing guanosines, i.e. guanosines from two adjacent strands in the same tetrad (9). Since one groove is made of several tetrads, to estimate its width the average distance over all its tetrads was calculated, as well as the standard deviation. The latter permits the evaluation of the variability of the distances within the groove. The P–P distance was shown to have an important variability, so C5’–C5’ and C3’–C3’ distances were also calculated (see Results and Discussion).

#### Calculation of the minimum groove width

**Figure 5.**
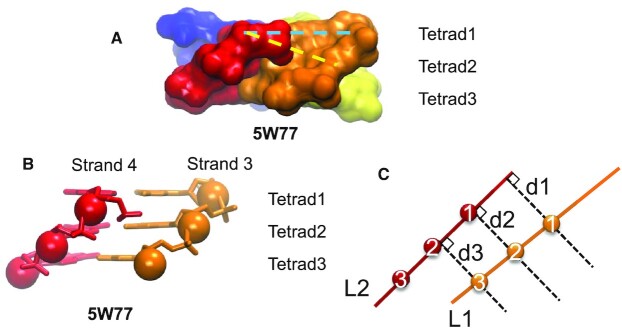
The space available in a groove. A medium groove from 5W77 is depicted as the solvent-accessible surface (**A**). The strands that delimit the groove are colored orange for strand 3, and red for strand 4. The C3’–C3’ distance in the same tetrad (blue dashed line) is bigger than the C3’–C3’ distance between tetrads 1 and 2 (yellow dashed line). (**B**) The same groove as (A), represented as sticks and spheres for atoms C3’. (**C**) 2D geometrical representation of (B). The two straight lines, L1 and L2, represent strands 3 and 4, respectively, and the spheres, atoms C3’. L1 is not strictly parallel to L2. Theoretically, to find the minimum distance between the short portions of L1 and L2, starting from L1, the perpendiculars to L2 must be drawn at different points and the shortest distances, d1, d2 and d3 calculated and averaged. The same can be done by descending the perpendiculars from L2 to L1. The result should be roughly similar. In the case of G4, there are no real continuous lines, but only discrete points along strands, which are the C3’ atoms. Normally, the shortest distances are the minimum C3’–C3’ distances calculated for each of the C3' of strand 3 (represented by L1). But, in G4, the strands are not long enough, so the shortest distance may not meet a C3’ atom, as for point 1 on L1, whose perpendicular does not meet any discreet point on L2. However, since our purpose is to estimate the average openness of the groove, the most appropriate way is to consider the average of the minimum C3’–C3’ distances calculated starting from strand 3. This calculation was also done starting from strand 4. As expected, the results were roughly the same, with differences well below the margin of error. This is why, for simplicity, ASC-G4 only provides the distances from the right strand to the left strand that delimit the groove.

Calculating the groove width generally aims to evaluate the space available to accommodate a lateral ligand or a protein segment, for instance. However, visual inspection of several structures convinced us that the C3’−C3’ or the C5’−C5’ distances of guanosines of adjacent strands in the same tetrad do not always reflect this available space, as shown in Figure 5A. This is either because the edges of the grooves are not parallel or, when they are almost parallel, because the strands are tilted relative to the tetrad planes. Consider the example of C3’; this applies as well to C5’. The C3’ atom of one guanosine, located in a given strand and a given tetrad, may be closer to the C3’ atom of one guanosine from the adjacent strand, but from another tetrad (Figure 5A). This narrows the groove width, and the space available to accommodate a potential ligand. Therefore, to estimate the real groove width, the distances were also calculated between the C3’ atom of one guanosine and the C3’ atoms of all the guanosines of the adjacent strand, and only the minimum distance was considered. This calculation was also done for the C5’ atoms. A more detailed explanation of this calculation is given in the legend of Figure 5. At this step, a file is provided that gives the identification of the nucleotides with the smallest C3’–C3’ and C5’–C5’ distances (Supplementary Figure S10). The minimum groove width is the average of these minimum distances calculated for the C3’ (or C5’) atoms of the groove. This better reflects the opening of the groove than the overall minimum because the groove can be wide open at one end and closed at another end. Both the groove widths and the minimum groove widths are given by ASC-G4.

#### Calculation of the backbone and sugar torsion angles

The backbone and sugar torsion angles of the entire nucleic chain that contains the G4 are given in the final output file (Supplementary Figure S11). The backbone angles, α, β, γ, δ, ϵ and ζ, and sugar angles, ν_0_, ν_1_, ν_2_, ν_3_ and ν_4_, are defined according to the IUPAC-IUB definition (12) as well as in Table 2–2 in (11). For the sugar, the phase angle of pseudorotation, *P*, and the degree of pucker, ψ_m_, are also provided. The definition of the latter two parameters is given on the website.

#### Determination of the multimer types and characteristics

Most intramolecular G4s are monomers, but some of them form dimers or are present as multimers in X-ray structures. We identified five multimer types: monomer, separate monomers, non-stacking-stem dimer, stacking-stem dimer, and interlaced dimer. (i) The monomer, as its name suggests, is an isolated nucleic chain, independently of the presence or not of a protein chain. (ii) The separate monomers are several juxtaposed monomers or dimers, or more generally, any two nucleic chains in the PDB file that do not have any stacking nts between them. (iii) The non-stacking-stem dimer corresponds to any two chains with nts, from out of the G4 stem, i.e. from either the loops or the 5’- or 3’-terminal tails, that stack over each other. (iv) The stacking-stem dimer consists of two G4s with two tetrads, one from each chain, stacking over each other. In this case, the two stems are a continuation of each other. (v) The interlaced dimer is a stacking-stem dimer with the particularity that in each of the two interface stacking tetrads, there is at least one nt from the other chain to complete the tetrad.

**Figure 6.**
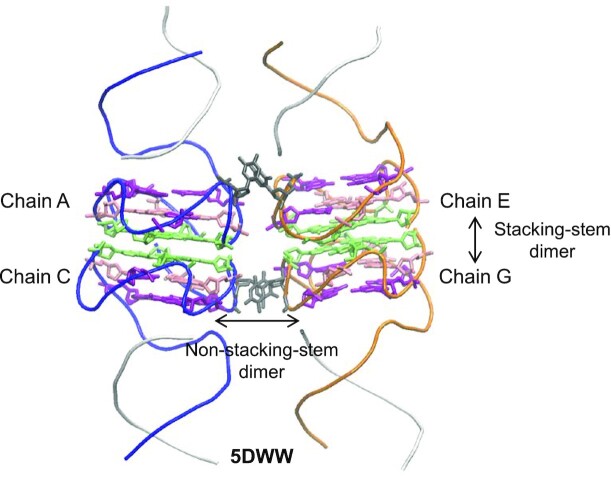
Example of different coexisting multimer types in structure 5DWW. In this multimer, there are two stacking-stem dimers: chains A–C (blue tubes) and chains E–G (orange tubes). In each stem, the tetrads are represented as sticks, colored according to the tetrad number: light green for tetrad 1, pink for tetrad 2, and purple for tetrad 3. In each chain, an additional nt from a loop is colored dark gray. These additional nts also stack over each other, resulting in two non-stacking-stem dimers: chains A–E and chains C–G. Conversely, chains A–G and chains C–E do not present any stacking nts, they are therefore qualified as separate monomers. The short chains B, D, F and H, which complete the Watson–Crick interactions with chains A, C, E and G, respectively, are drawn as white tubes.

Several types can coexist in the same PDB file. For instance, in 5DWW (36) (Figure 6), chains A and C form a stacking-stem dimer, as well as chains E and G. In addition to that, chains A and E, like chains C and G, form non-stacking-stem dimers, since there are stacking nts located in the loops. Finally, in the absence of any stacking nts, chains A and G, like chains C and E, are considered separate monomers.

In the case of a stacking-stem dimer or an interlaced dimer, i.e. types (iv) and (v), the characteristics of the interface are calculated, that is the rise of the guanines that form this interface, the tilt and twist angles, as well as the twist orientation (right- or left-handed).

The final output file of the program, which provides the most important structural characteristics of the G4, is presented in the Results and Discussion below.

### Graphics

All structural images of this article were done using the Visual Molecular Dynamics software (VMD) (37), and the plots, using Gnuplot on Windows system (http:/www.sourceforge.net/projects/gnuplot/).

## RESULTS AND DISCUSSION

### Program ASC-G4 in practice

The program can be used at the address http://tiny.cc/ASC-G4. For intramolecular G4, a structure file in the PDB format should be provided to detect and calculate all the features discussed above. However, if the coordinate file does not originate from the PDB, some information (like the chain, the sequence…), with the corresponding PDB keywords, should be added to make the program work properly. A template is provided on the website. When the structure is resolved by NMR, only the first frame is considered. In some X-ray structures, the electron density of some atoms reflects the possibility of two alternate positions. In this case, to be able to carry out the calculations, the coordinates are splitted in two separate files with the extension altlocA and altlocB. For intermolecular G4, the site is still under construction.

### The main output of the program

**Figure 7.**
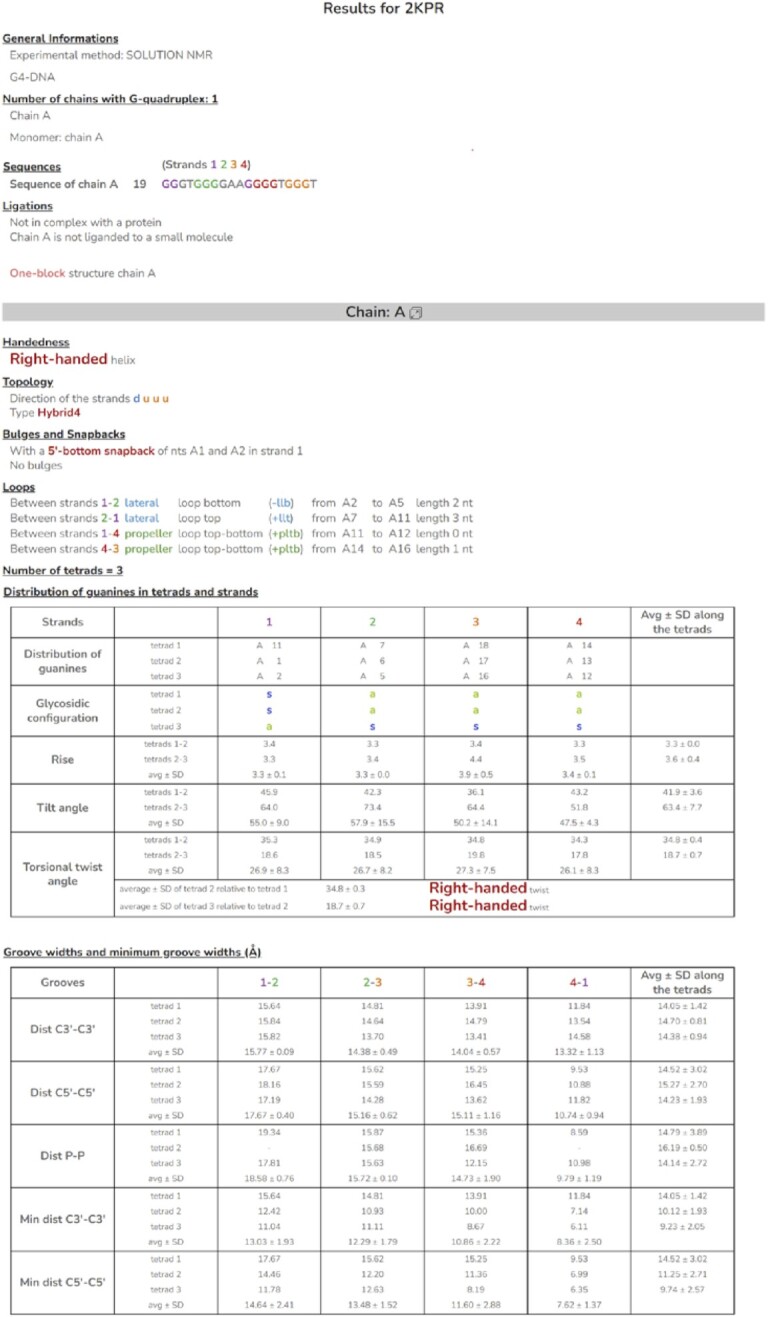
Main output file of ASC-G4.

For a given intramolecular G4 structure, the output of the program is provided in two formats, a colored HTML file, and a text file. It contains all the main structural characteristics. An example corresponding to the structure with PDB ID: 2KPR (14) is shown in Figure 7. It starts with some general information, like the experimental method used to resolve the structure (NMR or X-ray), the type of nucleic acid (DNA, RNA or hybrid DNA–RNA), the number of chains containing G4, the multimer types, the sequence, in which the guanines that are involved in strands are colored according to the strand number, the ligation state of the chains, i.e. the ligation with a protein and/or a small molecule, and finally, the type of the G4 (one block or two blocks). For the two-block structure, the tetrads, *n* and *n +*1, between which are located the three discontinuities that separate the two blocks are given. Block 1 always goes from tetrad 1 to tetrad *n* and block 2, from tetrad *n +*1 to the last tetrad. Please note that, in some cases, *n* can be equal to 1 and in other cases, *n +*1 can be equal to the last tetrad.

Then for each chain, the handedness of the helix (right-handed, left-handed or hybrid right-handed/left-handed) is given, followed by the direction of the strands (d for down and u for up) and the topology. When the G4 is made of two blocks, a separate topology type is attributed to each block. Then, follows the presence or absence of snapbacks and bulges, as well as the characteristics of the loops that connect the strands, i.e. their type (p for propeller, l for lateral, and d for diagonal), their length (the number of their nts), their position or direction (t for top and b for bottom), and their progression (‘+’ for the clockwise direction, when the G4 is oriented from bottom to top as described by da Silva (18), and ‘–’ for the anticlockwise progression). For the two-block structures, the list of linkers between the two blocks is also given (not applicable to the case of 2KPR).

Then, for each chain, there are two tables. The first one gives the distribution of the stem guanines in the tetrads (lines) and strands (columns), that is their chain and identification (position in the sequence). In the example of 2KPR, shown in Figure 7, the first tetrad consists of guanines A11, A7, A18, and A14, in strands 1, 2, 3, and 4, respectively (here A denotes the chain), and the first strand consists of guanines A11, A1 and A2, in tetrads 1, 2 and 3, respectively. By definition, the first strand is the one that contains the closest guanine to the 5’ end of the nucleic chain, except if this guanine corresponds to a 5’-top snapback (see the subsection entitled ‘Four different types of snapbacks’), in which case, the first strand is the one that contains the next guanines closest to the 5’ end. In the case of 2KPR, the first strand is made of guanines from two separate G-tracts, the first G-tract (A1 and A2) and the third G-tract (A11). This is reflected by the color code of the sequence (purple for strand 1). As observed, the first stem-guanine (in the one-block structure it is the guanine that belongs to both tetrad 1 and strand 1) is not A1, but A11, from the third G-tract (see also Figure 2B). The discontinuity in this strand between A1 and A11 shows the presence of a snapback, which we call here 5’-bottom (see the subsection entitled ‘Four different types of snapbacks’). Besides, the special orientation of the strands (duuu) classifies 2KPR in the hybrid4 topology (see the subsection entitled ‘Eight distinct topologies of the G4 structures’). In the first Table, are also given the gcs of the corresponding guanines. For instance, the guanines in the first tetrad of 2KPR, A11, A7, A18 and A14, have the gcs, s, a, a and a, respectively, where s stands for *syn* and a for *anti*. This is followed, for each strand and between two successive tetrads, by the rise of the guanine bases (i.e. the distance between the successive base planes), the tilt angles, and the torsional twist angles (see Materials and Methods for details). For each of these characteristics, their average (avg) along the tetrads and the strands, and standard deviation (SD) are given. Considering the torsional twist, the avg and SD of the angles along the tetrads represents the global rotation of one tetrad relative to the one above it. A positive average corresponds to a right-handed twist, and a negative average, to a left-handed twist. When, in a G4 stem, there is a mix of right-handed and left-handed twists, the global handedness is called hybrid right-handed/left-handed helix.

In the second Table, the columns do not correspond to the strands but to the grooves, designated by the number of the two strands that constitute the groove. It gives the distances between the C3’ atoms of every two guanosines that delimit the groove (i.e. that are located in two adjacent strands of the same tetrad), and the average C3’-C3’ distance along the strand, which is the groove width, and along the tetrad, in addition to their SD. Also, the C5’–C5’ distances and the P-P distances of adjacent strands are given, as well as their avg ± SD. This is followed by the minimum C3’–C3’ distances (i.e. the minimum distance between the C3’ atom of one guanosine and the C3’ atoms of all guanosines of the adjacent strand) and their avg ± SD. The average value along the strand is the minimum groove width. This is followed by the minimum C5’–C5’ distances. As observed for 2KPR, the difference between the groove width and the minimum groove width can be important, as for groove 4–1 (the fourth groove) this difference represents 37% of the groove width for C3’ and 29% for C5’.

When there are several chains, which is not the case with the monomer 2KPR, all the described characteristics are given for each chain. In the presence of a stacking-stem dimer or an interlaced dimer, the characteristics of the interface are also given, i.e. the identification of the stacking nts, their rise, tilt and twist angles, and their avg ± SD along the interface, as well as the orientation of one dimer relative to the other.

All this is followed by two other Tables (Supplementary Figure S11), where the torsion angles of the entire chain backbone (α, β, γ, δ, ϵ and ζ) and the sugar (ν_0_, ν_1_, ν_2_, ν_3_ and ν_4_) (11), as well as the sugar pseudorotation, *P*, and degree of pucker, ψ_m_, are also given. Besides, some additional information is also provided in some cases, by hovering the mouse over the text, to avoid ambiguities. This is the case, for instance, of the sequence when some nts are modified. If this modification is given explicitly in the PDB file under the keyword MODRES, the list is also given explicitly when hovering the mouse over the sequence, like for 4NI9 (38). However, in some cases, the modified nts are not specified in the PDB, so, they are given as M (for modified) in the sequence, like for 5MBR (39).

As described in the Materials and Methods, the information shown in Figure 7 is not the only one provided by the program. ASC-G4 also provides, in six other files, the distance between the heavy atoms involved in the Hoogsteen pairing, the distance between the O6 atoms and the distance of the O6 atom from the plane of its facing guanine (Supplementary Figure S3), the list of the stacking guanines, the distance between their C1’ atoms, the distance between the center of gravity of their bases, the angle between these base planes and their rise (Supplementary Figure S5), also the list of guanines that correspond to the minimum groove width (Supplementary Figure S10), and for each stem guanine, the value of its χ angle, the distances N3-O5’ and H1’-H8, as well as the distance of atom C1’ from the base plane (Supplementary Figure S7). Finally, for all the nts of the chain, the torsion angles of the backbone and the sugar are also given in two separate downloadable files. They are equivalent to the last two Tables on the website (Supplementary Figure S11). Please note that all information given on the website is also provided in a text format, to help the user in the analysis of the results.

### General information concerning our set of G4 structures

ASC-G4 was applied to the set of 207 G4 structures in a Linux loop, and their advanced structural characteristics were calculated in less than five minutes. 179 structures were DNA-G4s, 27, RNA-G4s, and one hybrid DNA–RNA-G4 (6FFR) (40). 70 structures were resolved using X-ray crystallography and 137 using NMR. 16 structures consisted of stacking-stem dimers (one tetrad from each chain stacked over each other) (Figure 6), one structure is an interlaced dimer (this structure is mostly an intramolecular G4, but where each of the two stacking-interface tetrads, i.e. tetrad 1 of both chains A and B, contains guanines from the two chains), 11 are non-stacking-stem dimers (the stacking nucleotides in the dimer are not part of the stem), 7 are separate monomers, (they are juxtaposed monomers without any stacking), and finally, 172 structures are simple monomers. Each chain in all these structures, except for the interlaced dimer, can be either made of one block or two separate blocks. The latter is observed when there are discontinuities, which are generally, but not necessarily, different from bulges or snapbacks, in at least three strands of two consecutive tetrads. In our set, 191 structures are made of one block and 15 structures, of two blocks. Both types of structures can form any type of the above-described multimers, except for the interlaced dimer.

196 G4 structures are right-handed helices and 5 are left-handed helices, whereas 6 are hybrid right-handed/left-handed helices (see Supplementary Tables S1 and S2 for details). Five of the latter structures are made of two blocks, which may explain the presence of the two different helix orientations, but the sixth structure, 2MCC (41), is a one-block structure, which is incompatible with these two helix orientations. On another hand, in a stacking-stem dimer or an interlaced dimer, whether each chain is a right-handed or a left-handed helix, the interface can be either right-handed or left-handed. In our set, nine dimers have a right-handed interface (all corresponding to right-handed monomers, of which 4 are two-block structures) and eight a left-handed interface (of which seven correspond to right-handed monomers and only one to left-handed monomers).

### ASC-G4 allows the assessment of the quality of the G4 structures

The information given by ASC-G4 can be used to assess the quality of the G4 structure. This is especially important for structures under construction. It also allowed us to assess the quality of the 207 structures of our set of study. As described in Materials and Methods, several G4s of this set presented structural issues. In two structures, 5EW1 and 5EW2 (42), in two guanines of one of the tetrads, atom N2 was positioned in the place of atom O6, toward the ions that are located between the tetrads, despite the low resolution of these structures (2.92 Å and 3.59 Å, respectively). Five structures contained Hoogsteen pairs with very loose H-bonds (distance between heavy atoms over 3.8 Å). 1OZ8 (26) had the highest number of loose Hbps (4 over 16 that constituted its G4 stem) and the biggest distance (4.91 Å) (Supplementary Figure S2A). Thirty-nine structures had non-coplanar Hoogsteen pairs of guanines, with a distance between atom O6 of one guanine and the base plane of its facing guanine between 1.00 and 2.8 Å (Supplementary Figures S12 and S2C). For atom names, refer to Figure 1F. 1MY9 (43) had the highest number of non-coplanar Hbps: 12 pairs over 16 that constituted its G4 stem dimer. Seven structures had non-parallel stacking guanines in their strands, i.e. with an angle between the base planes of two successive guanines of >25°. Unsurprisingly, 1MY9 had the highest number of non-parallel stacking pairs for successive guanines in three strands, and the biggest angle for one of them, 50.5°. In addition to that, nine structures, presented ambiguous stacking, i.e. one stem guanine stacks either between two strands or completely over a guanine from the adjacent strand instead of the guanine of its own strand (Supplementary Figure S4C).

Besides, the C1’ atom was either missing (5DWW, chain A, dG2) (36) or out of the base plane for 6.8 % of the stem guanines, which belonged to 69 structures. Supplementary Figure S6 shows the two extremes of these out-of-plane guanines: the one with the smallest distance of C1’ to the plane (N9–C4–C8), 0.15 Å, for 5NYT:dG4, and the one with the biggest distance, 0.80 Å, for 6SUU:dG4. Two NMR structures had all their stem guanines with C1’ out of the base plane: 2MCC (41) and 6SUU (44).

These issues did not seem to have any common reasons, like the resolution of the crystal structures or the force fields used for the refinement of the NMR structures (AMBER (parmbsc0, ff99bsc0, ff99bsc0 + GAFF + RESP charges), CFF in Discover/InsightII, or CHARMM in XPLOR). They probably originated from the individual treatment of each structure.

### Eight distinct topologies of the G4 structures

In ASC-G4, the first strand is the one that contains the first stem-guanine. By convention, it is oriented down (in a two-block structure, it is the segment that contains the first stem-guanine that is oriented down). The other strands are always numbered in the same orientation (Figure 3), following the gc of the first stem-guanine: since *anti*-G and *syn*-G are oriented in opposite directions, if the first stem-guanine is *anti*, the second strand is the one where the H-bond *donors* of this guanine point to, and if it is *syn*, the second strand is the one where the H-bond *acceptors* point to (see Materials and Methods for details). This numbering makes the strands unambiguously distinguishable.

Since the first strand is always down (d), there are 2^3^ = 8 possible combinations of strand directions, corresponding to eight different topologies: the five known topologies, which are given the same name as in the literature, including hybrid1 and hybrid2, in addition to three topologies that were never described, although they corresponded to existing structures; we called them hybrid3, hybrid4, and antiparallel-basket2. Please note that, although we used the top-to-bottom orientation of the G4 to better explain the principles of our classification method, and considered the first strand as always down to limit the redundant possibilities, this classification method is independent of the G4 orientation. Indeed, it is based on the ordering of the strands using the Hoogsteen interactions, which are intrinsic to the structure. In addition, although the absolute direction of the strands is dependent on the G4 orientation, this is not the case of their relative direction (one with respect to the others). Since the topology is determined by the relative direction of the strands which are ordered according to the Hoogsteen interactions, the topology is independent of the G4 orientation.

The distribution of the topologies of all the one-block structures, including the stacking-stem dimers, is given in Table 1. For each topology, the structures are dispatched according to the number of tetrads in the stem. For multimers, only the number of tetrads in one monomer is considered. As observed, the 3-tetrad G4s are predominant in the parallel and hybrid topologies, whereas the 2-tetrad G4s are predominant in the antiparallel topologies. A list of the PDB ID of all the one-block structures is given in Table S1.

**Table 1.** Distribution of the G4 topologies and their corresponding number of tetrads. The number of structures for each topology is given in column 2 and, for each topology, the number of the structures made of 2, 3 or 4 tetrads is given in columns 3, 4 and 5, respectively. The two-block G4s are omitted for clarity, leaving a total of 192 structures. For the strand directions, d stands for down and u for up

Topology (strand directions)	Number of structures	Number of structures with 2 tetrads	Number of structures with 3 tetrads	Number of structures with 4 tetrads
**Parallel** (dddd)	85	14	70	1
**Antiparallel-chair** (dudu)	37	33	2	2
**Antiparallel-basket** (duud)	19	10	5	4
**Antiparallel-basket2** (dduu)	12	9	2	1
**Hybrid1** (ddud)	12	0	11	1
**Hybrid2** (dddu)	11	4	7	0
**Hybrid3** (dudd)	11	1	10	0
**Hybrid4** (duuu)	5	0	5	0

In the literature, antiparallel-basket2 (25,45–51) is mixed up with antiparallel-basket, and hybrid3 (15,52–59) with hybrid2. This is because, usually, the strand numbering can be made in either anticlockwise or clockwise directions, making interchangeable the two strands that are adjacent to the first one, which creates topology ambiguities. Indeed, adopting both orientations amounts to interchanging strands 2 and 4. In this case, as shown in Supplementary Figure S13, the antiparallel-basket2 (with the strand directions dduu) would be equivalent to the antiparallel-basket (duud) by the inversion of strands 2 and 4, and similarly, hybrid3 (dudd) would be equivalent to hybrid2 (dddu). Regarding hybrid4, in the articles corresponding to each structure, the topology was either given as hybrid or it was not mentioned (16,60–62), except for structure 6R9L (63), which was quoted as hybrid1, due to the confusion in the hybrid specifications.

The originality of our classification method resides in the systematic numbering of the strands in the same orientation, based on the gc of the first stem-guanine. Recently, a new classification method called ONZ, using the ElTetrado program (64,65), was created to remove these ambiguities. In this method, each tetrad is first considered separately. Starting from the first guanine of the tetrad (with the lowest identification) the program follows the position in the sequence of the guanines that are in contact with it, either on its Watson–Crick (WC) edge (the guanine's H-bond donors) or on its Hoogsteen edge (the guanine's H-bond acceptors). The arrows that relate these guanines adopt the shape of the letters O, N, or Z, and therefore, determine the class attributed to this tetrad, i.e. O, N or Z. In addition, if the WC edge of the first guanine is oriented to the left, the tetrad is denoted +, otherwise, it is -. Then for the entire quadruplex, if all tetrads are of the same type (O, N or Z), the quadruplex is denoted by this type name, but if the tetrads are of different types, they are denoted M, as mixed. Then the topology is added, parallel (p), antiparallel (a) or hybrid (h). This ends with 10 possibilities, but in practice, only structures belonging to seven classes were found: Op, Oa, Oh, Na, Nh, Za and M. As can be seen, this classification, because it rotates in both clockwise (+) and anticlockwise (–) orientations, needed complex and unintuitive detours to remove ambiguities. Whereas it suffices to always adopt the same direction and attribute a number to each strand (1, 2, 3 and 4) to make them perfectly distinguishable, and therefore, remove all topology ambiguities.

### Topologies of the two-block structures

In a two-block structure, the first stem-guanine is also located in the first strand but it can be either in the first block or in the second block. The segment of the strand that contains the first stem-guanine is always oriented down. This determines the orientation of the other segment of the first strand (i.e. in the other block) which can, therefore, be either down or up. Consequently, in the two-block structures, there are two distinct topologies, one for each block. If the direction of the first strand is up in either the first or the second block, there are still eight distinct topologies for this block, but with reversed strand directions. For instance, when the directions are uuuu, the structure is parallel, when they are udud, it is antiparallel-chair, and so on. In our set of 207 G4s, there are 15 two-block structures, of which six are parallel/parallel; they are characterized by the relative orientation of their strands: dddd/dddd (1 structure), dddd/uuuu (4 structures), and uuuu/dddd (1 structure). For details see Table S2. There is also one parallel/hybrid2 structure with the strand directions uuuu/dddu. Besides, in some cases, one of the two blocks is made of only one tetrad, in which case, this block does not have any topology. Eight of the two-block structures are in this case, four of them are parallel/− and four are −/parallel. It should be noted that all the parallel/− could be at first glance considered to be one-block structures because their global view (Supplementary Figure S14A) does not show a clear separation as in other two-block structures (Supplementary Figure S1), and, contrary to other two-block structures, the discontinuities between the two blocks only consist of 1 to 2 nts bulges. However, on closer inspection, a subtler separation can be observed between the two blocks. Indeed, although all stem guanines are *anti*, in block 1 the bases are oriented to the left, and in block 2, to the right (Supplementary Figure S14B). This inversion of orientation, in addition to the presence of discontinuities in all four strands between tetrads 2 and 3, justify the division into two blocks.

### Four different types of snapbacks

**Figure 8.**
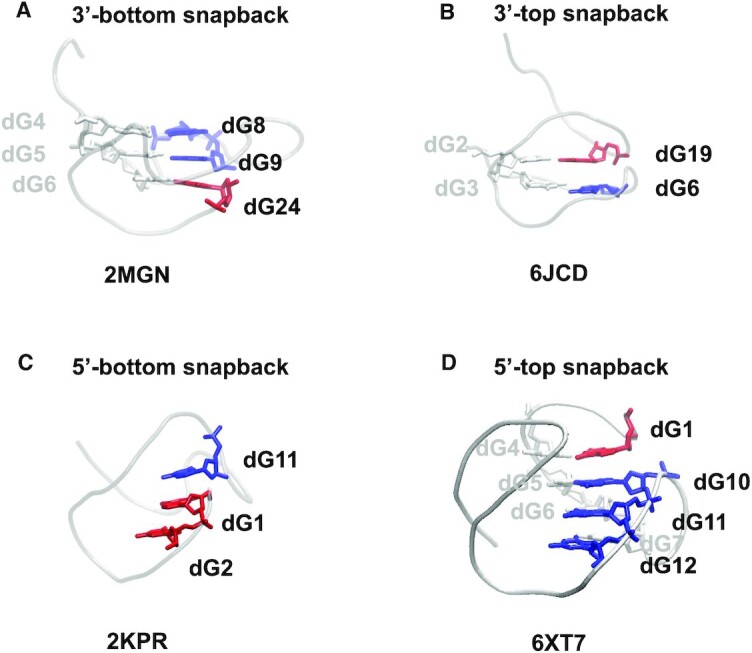
The different types of snapbacks. Each G4 structure is depicted as a light gray tube and only the strand where the snapback is located is shown as colored sticks, blue for the regular nts and red for the snapback. Contrary to (**C**), in (**A**), (**B**) and (**D**) the snapback is not located in the first strand, so, in these panels, the first-strand nts are depicted as light gray sticks to show the direction of the stem. The nucleotide identification is given near each nt and the PDB ID of the structure below its image.

Usually, only when the 3’-terminal guanine snaps back and inserts in the last tetrad at the bottom of the G4 stem that it is called a snapback. However, we observed the existence, by analogy, of other types of snapbacks, although they are not formally described, nor named as such. So, we expanded the definition to cover a more general case: a snapback is when either the 3’ or the 5’ extremity of the G4 stem snaps back to insert in either the bottom or the top tetrad(s). This definition results in four different possible types of snapbacks. Examples of these four types are reported in Figure 8: (A) the usual snapback that is called here 3’-bottom because it concerns the last guanine of the stem (or the last two guanines, as, for example, in 2O3M (66)) and it is located in the bottom tetrad(s); (B) the 3’-top snapback, which concerns the last guanine of the stem located in the top tetrad; (C) the 5’-bottom snapback, which concerns the first guanine (or two guanines) of the stem located in the bottom tetrad(s); (D) the 5’-top snapback, which concerns the first guanine of the stem located in the top tetrad. As shown in Table S1, which lists the one-block structures, nine were found to have a 3’-bottom snapback; they are all parallel. Six structures have a 5’-bottom snapback; two are parallel and four are hybrid4. Two structures have a 3’-top snapback, one is parallel and one is hybrid2. Finally, two structures have a 5’-top snapback; one is parallel and one is hybrid1. Therefore, it seems that the 3’-bottom snapback is characteristic of the parallel topology, and the 5’-bottom snapback is mainly characteristic of the hybrid4 topology. Nothing can be deduced for the other two types of snapbacks.

Regarding the two-block structures (Table S2), what is remarkable is that the 3’-bottom snapback is observed in all the left-handed parallel/parallel (dddd/uuuu) structures, and only in those. Three structures over 207 had 2 snapbacks, meaning that the two extremities of the stem are snapping back guanosines, one of these structures consists of one block, 6FQ2 (67), and two consist of two blocks, 6GZ6 (67) and 6KVB (62).

### Atoms C3’ and C5’ are more appropriate to calculate the groove width in G4 than atom P

**Figure 9.**
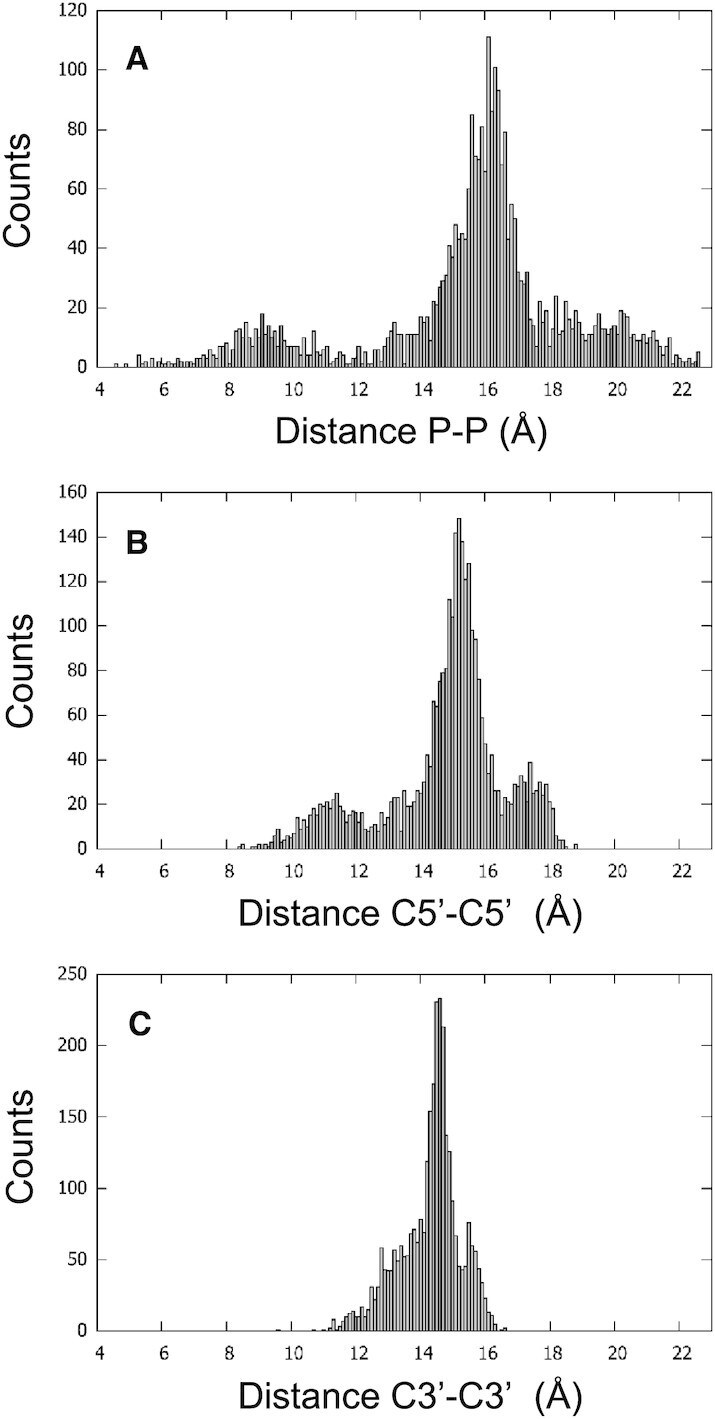
Distribution of the groove widths. The histograms are calculated for the individual P–P distances (**A**), C5’–C5’ distances (**B**) and C3’–C3’ distances (**C**), with a bin size of 0.1 Å.

Generally, the groove widths in G4 are not quantified, but, by analogy with dsDNA, they were evaluated for 2MS9, for instance, by calculating the distance between the phosphorus atoms (P) at the 5’-end of each two guanosines of adjacent strands (9). Therefore, to calculate the groove width here, we first considered the P − P distances. However, there were 77 structures over 207 in which the P atom of the first guanosine was missing, reducing the number of the groove widths that can be calculated, especially that each missing P impacts two grooves. In addition to this difficulty, an important variability of the P-P distances was observed within the grooves. To evaluate this variability, we considered the SD of this distance for each groove, then we calculated the average SD of the P-P distances over all the grooves. This average was < SD_P−P_> = 0.92 Å, with a maximum SD_P−P_ = 4.90 Å, showing a high variability of distances within the grooves. This makes the phosphorus atom not completely reliable for the groove width calculation of G4s. We, therefore, tried two other atoms from the backbone, C5’ and C3’. These atoms are present in all the grooves. For C5’, <SD_C5’−C5’_> = 0.56 Å, with a maximum SD_C5’−C5’_ = 2.70 Å, and for C3’, <SD_C3’−C3’_> = 0.33 Å, with a maximum SD_C3’−C3’_ = 2.00 Å. Since the C3’−C3’ distance had the smallest variability, it seemed the best for the calculation of the groove width, especially that the highest SD_C3’−C3’_ values were observed in structures with many issues, whereas the highest SD_C5’−C5’_ values were observed in structures with normal snapbacks and bulges. However, the comparison of the histograms of the P–P, C5’–C5’ and C3’–C3’ distances indicated that C5’ is the most appropriate for the calculation of the groove width (Figure 9). Indeed, whereas the P–P histogram presented a too-high range of distances (from 4.7 to 22.7 Å) and the C3’-C3’ histogram showed a continuous distribution with no clear separation between the narrow, medium, and wide grooves that are usually described in G4, the C5’–C5’ distribution showed three peaks, around 11.4, 15.2 and 17.4 Å.

Therefore, based on the distance variability within the grooves, C3’ atoms seem more appropriate for the calculation of the groove width, whereas based on the distance distribution, C5’ atoms seem more appropriate. Under these two considerations, P atoms do not seem well suited for the evaluation of the groove width in G4s. Anyway, it is up to the user to make his choice, provided that the name of the atom used for this evaluation is clearly specified in subsequent articles, to avoid ambiguities.

### Comparison between the groove width and the minimum groove width

**Figure 10.**
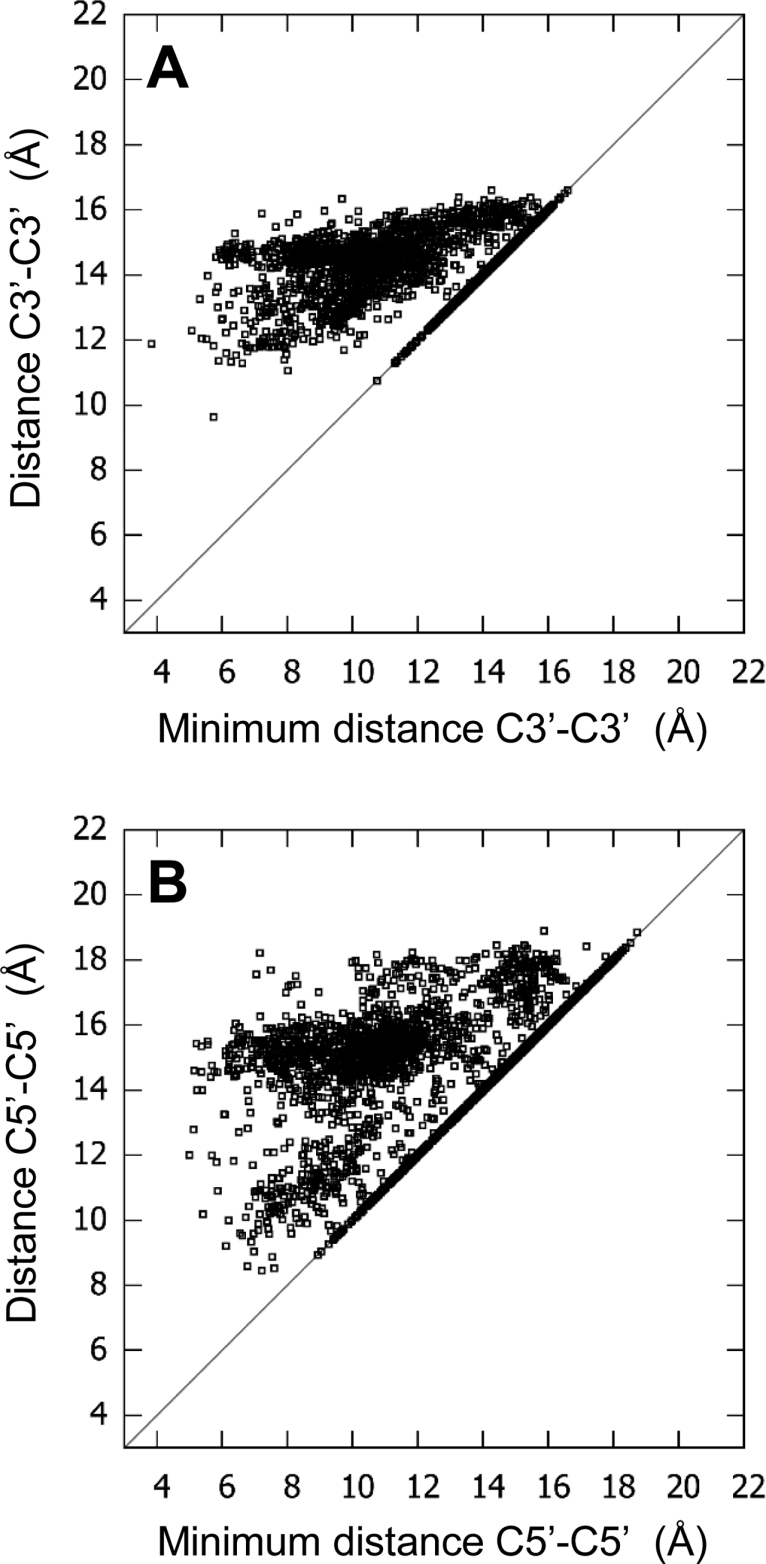
Distances C3’–C3’ (**A**) and C5’–C5’ (**B**) of Hbps versus the corresponding minimum distances C3’–C3’ and C5’–C5’, respectively.

Because the groove width does not always reflect the reality of the openness of the groove, due to the helix twist and tilt, the minimum groove width was also calculated, using atoms C3’ and C5’ (see Materials and Methods). The individual C3’–C3’ (or C5’–C5’) distances are reported *vs* the individual minimum C3’–C3’ (C5’-C5’) distances in Figure 10. This comparison shows that there is generally a great difference between the width and the minimum width, up to about 9 Å for C3’ and 11 Å for C5’. However, for both C3’ and C5’, 36–37% of the distances are equal to the minimum distances. This is mostly the case of the first tetrad (for 93% of these cases). The explanation can be given based on the schematic representation of the strands and the calculation of the minimum distance in Figure 5C, where the perpendicular line, d1, which is drawn for the calculation of this minimum for the first tetrad, does not meet any atom on the facing strand. Therefore, the minimum is the C3’–C3’ (or C5’–C5’) distance itself. Figure 10 reflects well that most grooves are less open than expected from their groove width calculation.

### When the range of the glycosidic bond angle is centered on 0° for *syn*-G and 180° for *anti*-G, the configuration is not satisfactory

To determine the glycosidic configuration (gc) of all stem guanines, their GBA, χ, was calculated, this angle being defined by the four atoms: O4’–C1’–N9–C4. In this subsection, the ranges, defined by the IUPAC-IUB (12) and by W. Saenger (11) were investigated. In these ranges, the configuration is considered *syn* for χ = 0 ± 90° and *anti* for χ = 180 ± 90°. W. Saenger (11) added the notions of *high-anti* for χ between −60° and −90°, which is described as part of *syn*, and *high-syn* for χ between 90° and 120°, which is described as part of *anti*. To compare these ranges with the experimental results of the G4s, the histogram of the χ angles was calculated for all the stem guanosines of our set of 207 structures. This histogram is bimodal, with two peaks at 60° and 240° (Figure 4 and Supplementary Figure S15A), which do not correspond to the center of the ranges adopted by the IUPAC-IUB for the *syn*-G and *anti*-G. Therefore, we shifted this histogram to fit it into these ranges (Supplementary Figure S15B). As observed, there is an important discrepancy between these ranges and the histogram since the tail of each unimodal distribution is cut and considered as part of the other configuration. This explains the need for the convoluted definitions of *high-anti*, which is part of *syn*, and *high-syn*, which is part of *anti*, to qualify them. Therefore, unsurprisingly, based on these ranges, some guanosines showed some inconsistency between the gc deduced from their GBA and the two other characteristics of *syn*-G and *anti*-G, i.e. the distances between atoms N3 and O5’, *d*_N3–O5’_, and atoms H1’ and H8, *d*_H1’–H8_. A short distance *d*_N3–O5’_ (<5.5 Å) indicates that the base faces the sugar as in *syn*-G, and a short distance *d*_H1’-H8_ (<3.2 Å) is representative, in NMR-resolved structures, of the presence of a NOESY cross-peak for also *syn*-G. Above these limits, the configuration is considered as *anti*. The limit distances of *d*_N3–O5’_ and *d*_H1’–H8_ were defined here based on structural observations. Two examples illustrate the inconsistency between GBA and these distances. 2M6W:dG7 (Supplementary Figure S16A), χ = 99°, presumably indicates an *anti* configuration, according to the IUPAC-IUB definition, whereas the base faces the sugar, with a very short distance N3-O5’, *d*_N3-O5’_ = 2.7 Å, and experimentally a NOESY signal is observed between H1’ and H8 (68), (*d*_H1’–H8_ = 2.7 Å), as in a *syn* configuration. Conversely, for 1C35:dG2 (17) (Supplementary Figure S16B), χ = –74°, presumably corresponds to a *syn* configuration, according to the IUPAC-IUB definition, whereas *d*_N3–O5’_ = 6.3 Å and *d*_H1’–H8_ = 3.9 Å indicate an *anti* configuration. For the stem guanosines, the ranges of the χ angle adopted by the IUPAC-IUB to define the *syn* and *anti* configurations are contradictory with the experimental observations of NOESY cross-peaks for 5.3% of these guanosines in our set of NMR-resolved structures. These ranges are also contradictory with the N3–O5’ distance values for 6.0 % of the stem guanosines in all our set of structures, although this distance is usually presented as the consequence of the χ angle value.

The observed discrepancy between the histogram of GBA and the IUPAC-IUB definition of *syn*-G and *anti*-G, only concerns the stem guanosines in G4, to which this study is limited. Even for these guanosines, we wondered if this discrepancy could not be due to the structure resolution. Indeed, 2/3 of the structures in our set were resolved by NMR, which is combined with molecular dynamics simulations (MD) under restraints for structure refinement, and therefore, the gc distribution could be due to these restraints or the force fields used in MD. In NMR, if high-intensity NOESY cross-peaks are observed between atoms H1’ and H8 of stem guanosines, their configuration is considered to be *syn*, otherwise, it is *anti*. The translation of this information is a restraint for the MD simulations, which is not always described in the corresponding articles. However, when it is described, it is usually introduced as a torsion angle restraint on χ, with the reference χ angle mostly centered on 60° for *syn*-G and 240° for *anti*-G (14,15,60,69–71) with more or less large intervals. But in some scarce cases, it was centered on 0° for *syn*-G and either on 135° (68) or 180° (16) or 240° (72) for *anti*-G. All these structures ended, after MD refinement, in the zones centered on 60° for *syn*-G and 240° for *anti*-G. This is even the case for the guanosines that were restrained at 0° for *syn*-G and 180° for *anti*-G because the restraints were loose enough to allow this modification. For structures that ended in the indeterminacy intervals, the reference χ angle was generally not specified in the corresponding articles. Therefore, the distribution of the χ angles does not seem to result from the restraints. However, it could be due to the force fields used in MD simulations of NMR structures. This neither does not seem to be the case since, in the crystal structures, which are not generally submitted to MD simulations, the glycosidic torsion angles are also centered on 60° and 240°. The distribution of configurations observed here is also in good agreement with the theoretical results, obtained by quantum mechanics calculations on deoxyguanosine, published by Foloppe *et al.* (13). Therefore, these intervals give more confidence and must be adopted without ambiguity.

### The glycosidic configuration with the ranges of χ centered on 60° for *syn*-G and 240° for *anti*-G is coherent with the distances *d*_H1’–H8_ and *d*_N3–O5’_

**Figure 11.**
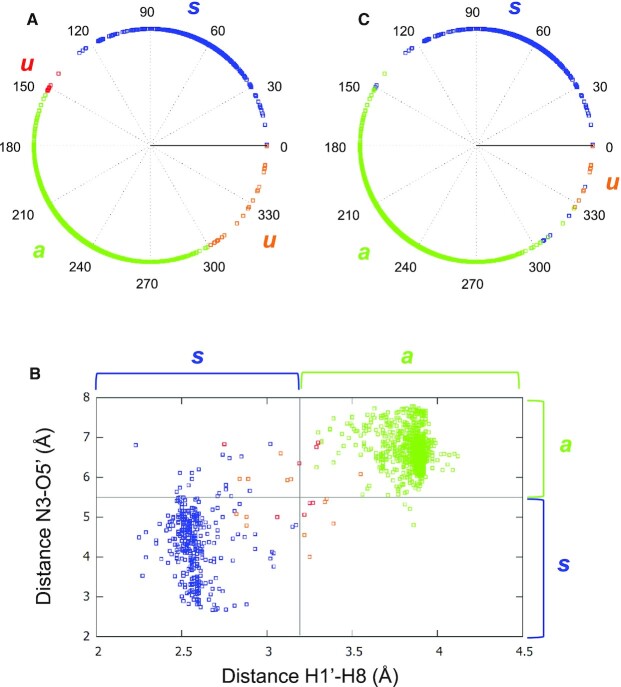
The glycosidic configurations. (**A**) The $\chi$angles of the stem guanosines are reported on a circle and colored according to the histogram ranges in Figure 4: blue for ${\rm{\chi }} \in {] {0^\circ ,140^\circ } ]}$, green for ${\rm{\chi }} \in {] {152^\circ ,300^\circ } ]}$, red for ${\rm{\chi }} \in {] {140^\circ ,152^\circ } ]}$, and orange for ${\rm{\chi }} \in {] {300^\circ ,360^\circ } ]}$. (**B**) Distance N3–O5’ versus distance H1’–H8 (for the atom names refer to Figure 1F). The gray lines delimit the *syn* and *anti* regions for each of these distances, as also shown with the brackets to the right and on the top of the plot. These limits are 5.5 Å for distance N3-O5’ and 3.2 Å for distance H1’–H8. The square points are colored according to the $\chi$angle and its color code in (A). (**C**) The$\chi$angles of the stem guanosines are reported on a circle as in (A) but colored according to the final gcs: blue for *syn-G*, green for *anti*-G, and orange for the undetermined gcs. The difference between (A) and (C) is the color code, which represents GBA in (A) and gc in (C).

The histogram of the χ angles is a linear representation of a circular variable, hence the need of shifting it when changing the ranges. We, therefore, preferred to adopt a circular representation, by reporting the GBA, χ, calculated for all the stem guanosines of the 207 G4 structures, on a circle (Figure 11A). As in the bimodal histogram (Figures 4 and Supplementary S14A), two densely populated zones were observed, centered on 60° for *syn*, and 240° for *anti*. More precisely, the configuration seemed to be *syn* for ${\rm{\chi }} \in {] {0^\circ ,140^\circ } ]}$ and *anti* for ${\rm{\chi }} \in {] {152^\circ ,300^\circ } ]}$. As a reminder, the mathematical notation ${\rm{\chi }} \in {] {0^\circ ,140^\circ } ]}$ is equivalent to 0° < χ ≤ 140°. Between these two zones, there were two regions, a narrow one in the range ]140°,152°] (in red in Figure 11A), and a wide one in the range ]300°,360°] (in orange in Figure 11A), that are more sparsely populated, where the configuration was undetermined, i.e. it might be either *syn* or *anti*.

As presented in the previous subsection, the configuration can also be described using both distances *d*_H1’–H8_ and *d*_N3–O5’_. Therefore, we reported *d*_N3–O5’_ versus *d*_H1’–H8_ in Figure 11B. In the plot, which mostly concerns the NMR structures since only two X-ray structures have H-atoms, the square points were colored according to the predicted configuration, based on the χ angle with the ranges that we finally adopted, i.e. centered on 60° for *syn*-G and 240° for *anti*-G. With these ranges, there is a full agreement between the configuration based on the χ angle and the distance *d*_H1’-H8_, if the limit distance between *syn*-G and *anti*-G is 3.2 Å. There is also a substantially better agreement with *d*_N3–O5’_ compared to the IUPAC-IUB (12) ranges where the χ angle was centered on 0° for *syn*-G and 180° for *anti*-G. Indeed, for all structures (crystal and NMR), when considering the determined configurations (i.e. with χ in the ranges ]0°,140°] for *syn* and ]152°,300°] for *anti*), the discrepancies between the χ angle and the distance *d*_N3–O5’_ are observed for <0.9 % of the stem guanosines, to be compared with the 6% discrepancies observed before.

### Removing the indeterminacy of the glycosidic configuration when possible

Considering the undetermined configurations based on the χ angle, we relied on the distances *d*_N3–O5’_ and *d*_H1’–H8_ to remove this indeterminacy. However, this can only be done when C1’ is in the base plane, i.e. for the eight guanosines in the narrow range, ]140°,152°] (red points in Figure 11A), and fifteen of the guanosines in the wide range, ]300°,360°] (orange points in Figure 11A). For the other eleven guanosines in the wide range, ]300°,360°], the indeterminacy remains because C1’ is out of the base plane. Therefore, there is a total of 23 undetermined-configuration guanosines with the right χ angle (i.e. with C1’ in the base plane), for which the indeterminacy can be removed. As observed in Figure 11B, only seven of these guanosines have a good agreement between the configuration determined from the two distances *d*_N3–O5’_ and *d*_H1’–H8_. They correspond to the red and orange points located in the upper-right (*anti*-G) and lower-left (*syn*-G) quadrants of the figure panel, and therefore, their configuration is deduced from this location. For the other 16 guanosines, they are located in the discrepancy quadrants, that is in the lower-right and the upper-left sections, meaning that the distances *d*_N3–O5’_ and *d*_H1’–H8_ indicate opposite configurations. The remoteness from the limits, $| {{r}_{{\rm{N3 - O5^{\prime}}}}} |$and $| {{r}_{{\rm{H1^{\prime} - H8}}}} |$ (see Materials and Methods for details), was used to determine the configuration of these guanosines. The final gcs are reported in Figure 11C. We observe that in both the indeterminacy regions, the final configurations are mixed between *syn* and *anti*, showing that it is not possible to put a clear limit between the *syn* and *anti* configurations, only based on GBA. Eventually, in our set of structures, there were 2213 *anti*, 595 *syn* and 11 undetermined configurations.

As observed, the determination of the gc is not always straightforward and can be complex in some cases, hence the use of distances, *d*_N3–O5’_ or *d*_H1’–H8_, to determine some glycosidic configurations. Because of this, here, we make a distinction between the glycosidic bond angle (GBA) and the glycosidic configuration (gc).

## CONCLUSION

This study describes the program ASC-G4, and the corresponding website, which allows, in addition to the usual structural features, the calculation of many G4 characteristics that are not available elsewhere, like the tilt angle, the groove width, and the minimum groove width. The analysis of the results shows that, by simply numbering the strands in always the same direction, the ambiguities of the G4 topologies are naturally removed, and the hybrid structures, for instance, become easily distinguishable. Four different types of snapbacks are found in structures, the 3’-bottom seems to be characteristic of the parallel topology and the 5’-bottom mainly of the hybrid4 topology. Nothing can be concluded for the other two snapbacks, 5’-top and 3’-top. In addition to that, the choice of the atoms to use for the calculation of the groove width is rationalized by comparing the variability of this width along the groove as well as the distribution of the groove widths. It happened that the C3’ atom presents the least variability, whereas C5’ presents a 3-peak distribution. Therefore, both can be adopted for the groove width calculation. However, the grooves are shown to be less open than their widths suggest, as can be concluded from the comparison between the C3’–C3’ (C5’–C5’) distances and the minimum C3’–C3’ (C5’–C5’) distances. On another hand, it is shown that there is no consensus about the correspondence between the glycosidic configuration (gc) and the value of the glycosidic bond angle (GBA or χ), and that, in some cases, the determination of the gc is complex and cannot be simply reduced to GBA, hence the need of characteristic distances to help remove some indeterminacy. Besides, ASC-G4 can help assess the quality of G4 structures, by providing supplementary structural information in several downloadable files.

## DATA AVAILABILITY

The ASC-G4 website is at the following address: http://tiny.cc/ASC-G4.

## Supplementary Material

gkad060_Supplemental_File

## Notes

Present address: Marc Farag, Normandie Université, Unicaen, CERMN, F-14000 Caen, France.
