# Supplementary material for: ASC-G4, an algorithm to calculate advanced structural characteristics of G-quadruplexes
Source: Nucleic Acids Res. 2023 Feb 16;51(5):2087–107. doi: 10.1093/nar/gkad060 (PMC10018348; doi:10.1093/nar/gkad060)
Supplement: gkad060_Supplemental_File [file gkad060_supplemental_file.pdf]

SUPPLEMENTARY DATA

ASC-G4, an algorithm to calculate advanced structural characteristics of G-quadruplexes

Marc Farag, Cédric Messaoudi, and Liliane Mouawad*

CONTENTS

p. 2 Figure S1. A G4 structure made of 4 tetrads separated into two blocks.

p. 3 Figure S2. Structural issues for the detection of tetrads.

p. 4 Figure S3. Output file of distances.

p. 5 Figure S4. Structural issues for the detection of strands.

p. 6 Figure S5. Output file of stacking nucleotides of structure 2KPR.

p. 7 Figure S6. The two extreme guanosines with the C1' atom out of the base plane.

p. 8 Figure S7. Output file of configurations.

p. 9 Figure S8. Part of the results of ASC-G4 for 7D5E.

p. 10 Figure S9. The torsional twist angle and the tilt angle.

p. 11 Figure S10. Output file of the minimum groove widths.

p. 12 Figure S11. Output of the main chain and sugar torsion angles.

p. 13 Table S1. Distribution of the topologies of the 192 one-block and interlaced structures.

p. 14 Table S2. Distribution of the topologies of the 15 two-block structures.

p. 15 Figure S12. The two extreme non-coplanar Hoogsteen base-pairs of guanines.

p. 16 Figure S13 (part 1). Investigation of the difference in the topologies with both clockwise and anticlockwise orientations of the strand numbering.

p. 17 Figure S13 (part 2). Same as S13 (part 1) but for hybrid topologies.

p. 18 Figure S14. Representative structure of a two-block parallel/− topology, 6C64.

p. 19 Figure S15. Histogram of GBA depicted according to two different ranges.

p. 20 Figure S16. Illustration of the discrepancies between the different characteristics of the configuration.

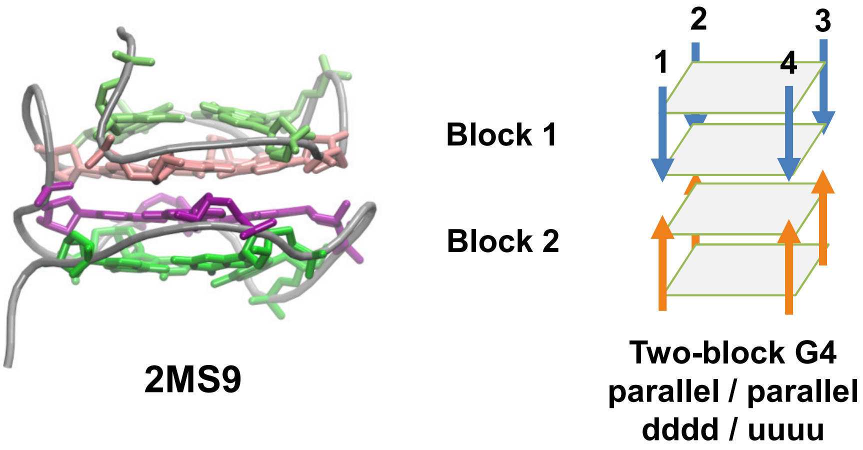

Figure S1. A G4 structure made of 4 tetrads separated into two blocks. Left panel: structure PDB ID 2MS9, with the following color code, 1st tetrad in light green, 2nd tetrad in pink, 3rd tetrad in purple, and 4th tetrad in bright green; the rest is a gray tube. Right panel: schematic representation of 2MS9 where the tetrads are represented as gray planes and the strands as blue arrows for down, and orange arrows for up. The bold numbers on the top are the strand numbers and the topologies below are those of the two blocks. In this case, the two blocks are parallel but in opposite directions. Block 1 consists of tetrads 1-2 and block 2, of tetrads 3-4.

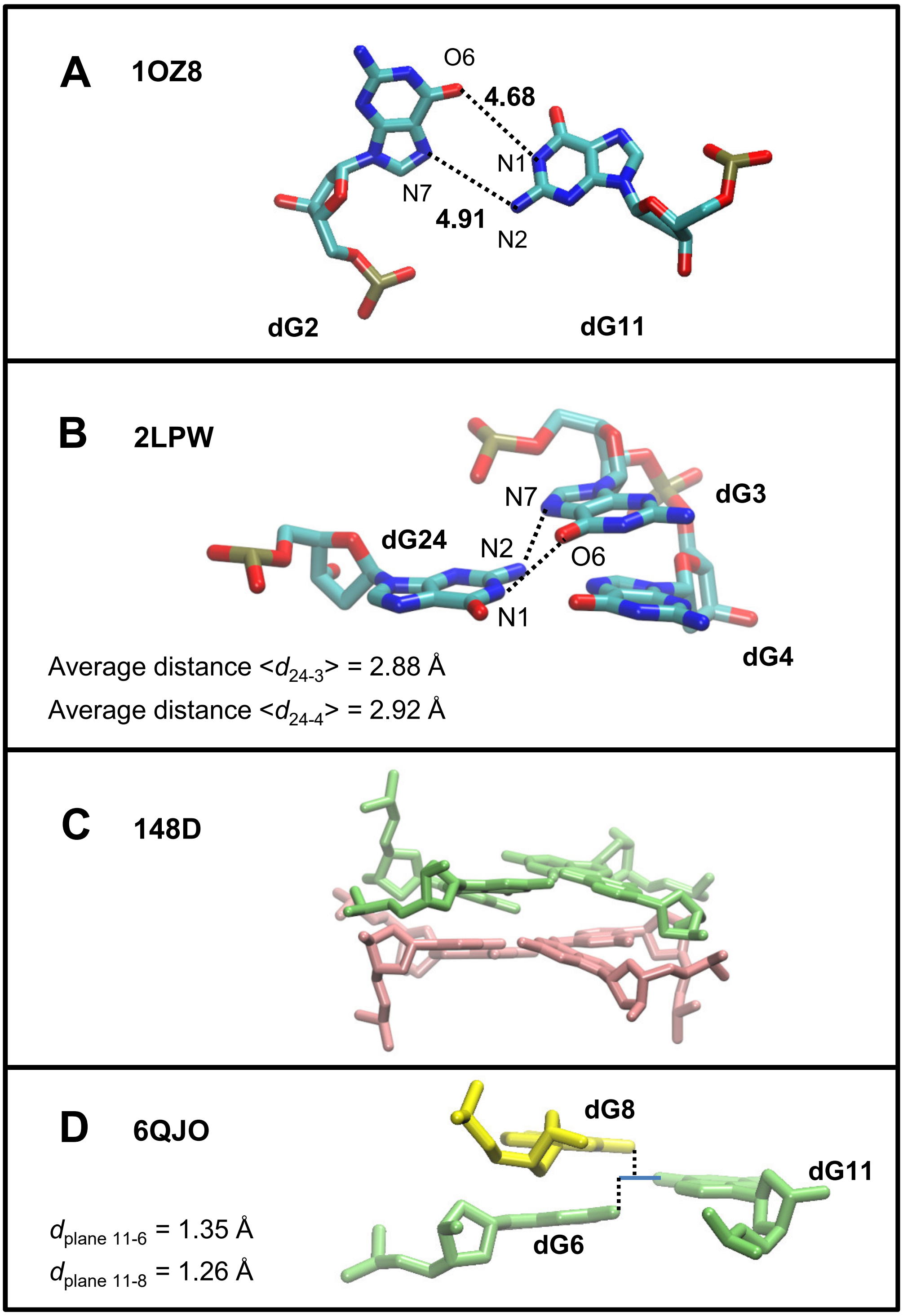

Figure S2. Structural issues for the detection of tetrads. (A) Very loose H-bonds in a Hoogsteen base-pair in structure 1OZ8. (B) The example of 2LPW illustrates the case where a guanine of a tetrad (here dG24) is closer to the facing guanine from another tetrad (dG3) than to the facing guanine of the same tetrad (dG4). The color code of atoms is as follows: C (cyan), N (blue), O (red), and P (tan). (C) The two tetrads of 148D are drawn, the first in light green and the second in pink. As observed they are not planar. (D) In 6QJO, chain B, the distance between the planes of dG11 and dG6 is greater than the distance between dG11 and dG8, although dG6 and dG11 are part of the same tetrad (light green), and dG8 (yellow) is in a loop. The blue plain line represents the base plane and the black dashed line the distance of O6 to this plane. Hydrogen atoms are omitted for clarity.

```
A 1 N1       A 13 O6          dist1 = 2.95912
A 1 N2       A 13 N7          dist2 = 3.05447  aver_dist = 3.0068
A 1 O6       A 13 O6          distO6-O6  = 3.36509
distance to plane[A 1,A 13] = 0.0854356 A
A 2 N1       A 5 O6          dist1 = 2.99158
A 2 N2       A 5 N7          dist2 = 2.94498  aver_dist = 2.96828
A 2 O6       A 5 O6          distO6-O6  = 3.11403
distance to plane[A 2,A 5] = 0.340549 A
A 5 N1       A 16 O6          dist1 = 3.03149
A 5 N2       A 16 N7          dist2 = 2.93444  aver_dist = 2.98297
A 5 O6       A 16 O6          distO6-O6  = 3.25481
distance to plane[A 5,A 16] = 0.098942 A
A 6 N1       A 1 O6          dist1 = 3.02651
A 6 N2       A 1 N7          dist2 = 3.0263  aver_dist = 3.02641
A 6 O6       A 1 O6          distO6-O6  = 3.37961
distance to plane[A 6,A 1] = 0.118005 A
A 7 N1       A 11 O6          dist1 = 3.01814
A 7 N2       A 11 N7          dist2 = 2.97658  aver_dist = 2.99736
A 7 O6       A 11 O6          distO6-O6  = 3.20373
distance to plane[A 7,A 11] = 0.258203 A
A 11 N1       A 14 O6          dist1 = 3.03009
A 11 N2       A 14 N7          dist2 = 2.96115  aver_dist = 2.99562
A 11 O6       A 14 O6          distO6-O6  = 3.30195
distance to plane[A 11,A 14] = 0.345805 A
A 12 N1       A 2 O6          dist1 = 3.0173
A 12 N2       A 2 N7          dist2 = 2.98948  aver_dist = 3.00339
A 12 O6       A 2 O6          distO6-O6  = 3.31576
distance to plane[A 12,A 2] = 0.0252199 A
A 13 N1       A 17 O6          dist1 = 2.99282
A 13 N2       A 17 N7          dist2 = 2.99102  aver_dist = 2.99192
A 13 O6       A 17 O6          distO6-O6  = 3.40476
distance to plane[A 13,A 17] = 0.0974183 A
A 14 N1       A 18 O6          dist1 = 2.9915
A 14 N2       A 18 N7          dist2 = 3.00883  aver_dist = 3.00016
A 14 O6       A 18 O6          distO6-O6  = 3.11632
distance to plane[A 14,A 18] = 0.256348 A
A 16 N1       A 12 O6          dist1 = 2.97976
A 16 N2       A 12 N7          dist2 = 3.02274  aver_dist = 3.00125
A 16 O6       A 12 O6          distO6-O6  = 3.09894
distance to plane[A 16,A 12] = 0.215688 A
A 17 N1       A 6 O6          dist1 = 2.99491
A 17 N2       A 6 N7          dist2 = 3.03345  aver_dist = 3.01418
A 17 O6       A 6 O6          distO6-O6  = 3.40905
distance to plane[A 17,A 6] = 0.164951 A
A 18 N1       A 7 O6          dist1 = 3.00886
A 18 N2       A 7 N7          dist2 = 3.02979  aver_dist = 3.01933
A 18 O6       A 7 O6          distO6-O6  = 3.33816
distance to plane[A 18,A 7] = 0.220188 A

# list of facing guanines          distC3'    distC5'    distP
    A 1         A 13               13.542     10.883      0.000
    A 2         A 5                15.824     17.189     17.813
    A 5         A 16               13.698     14.278     15.627
    A 6         A 1                15.837     18.161      0.000
    A 7         A 11               15.636     17.668     19.341
    A 11        A 14               11.845      9.528      8.592
    A 12        A 2                14.584     11.824     10.981
    A 13        A 17               14.792     16.455     16.689
    A 14        A 18               13.912     15.247     15.362
    A 16        A 12               13.415     13.622     12.153
    A 17        A 6                14.640     15.588     15.677
    A 18        A 7                14.814     15.619     15.867
end_of_list
```

Figure S3. Output file of distances. Upper part: Hoogsteen distances, d_1, d_2, < d >, $d_{O6\text{-}O6}$ and distance to plane[i,j]. The latter corresponds to the distance from O6 of the H-bond acceptor [j] to the plane of the H-bond donor [i] defined by atoms, N1, O6, and N7. Lower part: list of the facing guanines and their C3'-C3', C5'-C5', and P-P distances. This file corresponds to structure 2KPR.

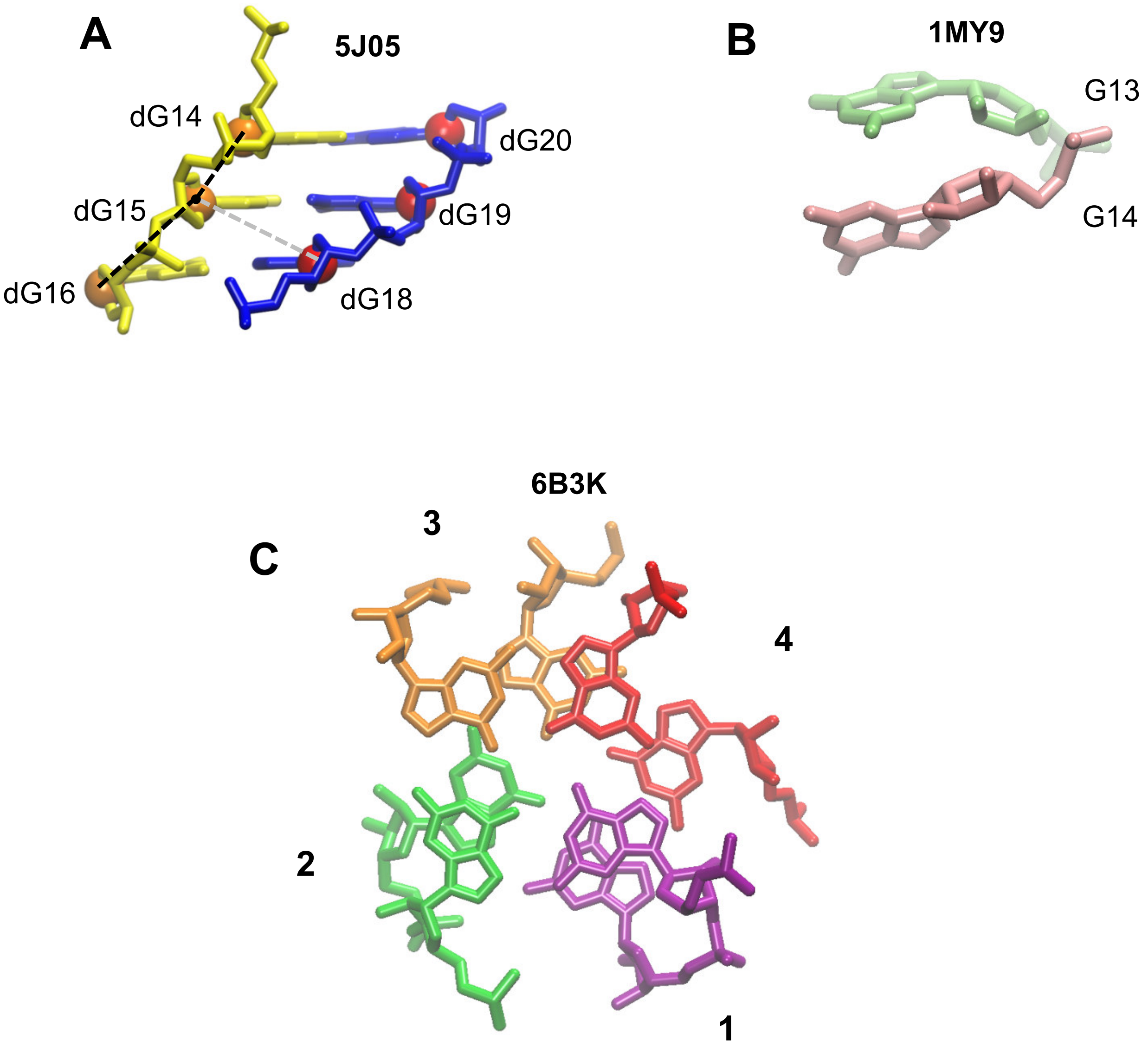

Figure S4. Structural issues for the detection of strands. (A) Two adjacent strands (the penultimate and ultimate ones) of 5J05 are represented as yellow and blue sticks. The C1' atoms are orange and red spheres. Normally, the C1'-C1' distance between successive guanines of the same strand (dashed black lines) should be smaller than between two adjacent strands (dashed gray line), whereas here, $d_{C1'(15)-C1'(18)} < d_{C1'(15)-C1'(16)}$. (B) Two stacking guanines in a strand. They are expected to be parallel, but the plane-plane angle is equal to 50.5°. (C) Structure 6B3K consists of two tetrads. Each strand is colored and numbered differently. As can be observed, whereas in strands 1 and 2 (purple and green, respectively) guanines of the top tetrad stack well over those of the bottom tetrad, in strands 3 and 4 (orange and red, respectively) the upper guanines are shifted relative to the lower ones. Therefore, the upper G of strand 3 stacks over nothing, and that of strand 4, stacks over the lower G of strand 3, leaving the lower G of strand 4 without an upper stack.

#	Nucleotides	distC1'-C1'	distCG1-CG2	cos(base-plane-angle)	rise
A	1 DG - A 2 DG	4.84	4.28	-0.9978	3.2924
A	1 DG - A 11 DG	5.97	4.44	0.9987	3.4062
A	2 DG - A 3 DG	4.57	3.71	0.9963	3.2334
A	5 DG - A 6 DG	4.10	3.71	-0.9977	3.3569
A	6 DG - A 7 DG	5.55	4.38	0.9925	3.2737
A	7 DG - A 8 DG	4.74	3.93	0.9869	3.7876
A	10 DA - A 11 DG	3.66	4.70	-0.9547	3.8701
A	12 DG - A 13 DG	5.38	4.48	-0.9936	3.4816
A	13 DG - A 14 DG	5.84	4.80	0.9920	3.2742
A	16 DG - A 17 DG	5.21	4.52	-0.9756	4.3684
A	17 DG - A 18 DG	6.03	4.66	0.9951	3.3690
A	18 DG - A 19 DT	5.46	3.97	-0.9346	3.6931

Figure S5. Output file of stacking nucleotides of structure 2KPR. CG is the center of gravity of the purine heavy atoms of G, A, and I, and of the pyrimidine heavy atoms of C, T, and U. Cos (base-plane-angle) is the cosine of the angle between the base planes of the two stacking nucleotides. The rise is the distance between the two base planes.

A

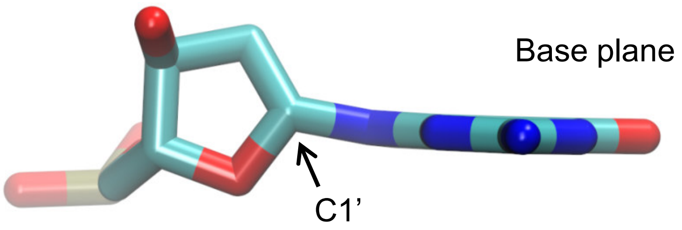

5NYT:dG4

Distance of C1’ to plane (N9-C4-C8) = 0.15 Å

B

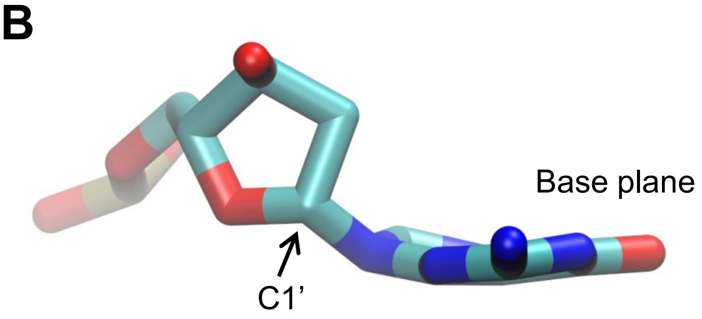

6SUU:dG4

Distance of C1’ to plane (N9-C4-C8) = 0.80 Å

Figure S6. The two extreme guanosines with the C1’ atom out of the base plane. (A) 5NYT:dG4 has the smallest distance (C1’-base plane) visually detectable. (B) 6SUU:dG4 has the largest distance (C1’-base plane) of our set of G4 structures. The atoms' color code is C (cyan), N (blue), O (red), and P (tan). Hydrogen atoms are omitted for clarity.

```
Dihedral angle (O4'-C1'-N9-C4), configuration of GUA: syn (s), anti (a), and undetermined (u)
distance N3-O5', distance H1'-H8, dist of atom C1' to plane N9-C4-C8, and final confguration
=========================================================================================
nucid =  A 1      chi angle =   53.50  s      dist N3-O5' =  3.09  s      dist H1'-H8 =  2.53  s      dist_to_plane =  0.0102566        s
nucid =  A 2      chi angle =  245.62  a      dist N3-O5' =  6.43  a      dist H1'-H8 =  3.89  a      dist_to_plane =  0.0476204        a
nucid =  A 5      chi angle =   90.02  s      dist N3-O5' =  4.85  s      dist H1'-H8 =  2.61  s      dist_to_plane =  0.0164290        s
nucid =  A 6      chi angle =  230.91  a      dist N3-O5' =  6.65  a      dist H1'-H8 =  3.90  a      dist_to_plane =  0.0237471        a
nucid =  A 7      chi angle =  241.54  a      dist N3-O5' =  6.35  a      dist H1'-H8 =  3.90  a      dist_to_plane =  0.0292652        a
nucid =  A 11     chi angle =   43.61  s      dist N3-O5' =  4.63  s      dist H1'-H8 =  2.55  s      dist_to_plane =  0.0504835        s
nucid =  A 12     chi angle =  322.84  u      dist N3-O5' =  4.84  s      dist H1'-H8 =  3.39  a      dist_to_plane =  0.0392724        s
nucid =  A 13     chi angle =  230.06  a      dist N3-O5' =  7.00  a      dist H1'-H8 =  3.90  a      dist_to_plane =  0.1015457        a
nucid =  A 14     chi angle =  234.89  a      dist N3-O5' =  6.47  a      dist H1'-H8 =  3.91  a      dist_to_plane =  0.0947781        a
nucid =  A 16     chi angle =   50.14  s      dist N3-O5' =  3.36  s      dist H1'-H8 =  2.61  s      dist_to_plane =  0.0249203        s
nucid =  A 17     chi angle =  248.33  a      dist N3-O5' =  6.43  a      dist H1'-H8 =  4.00  a      dist_to_plane =  0.0438885        a
nucid =  A 18     chi angle =  206.47  a      dist N3-O5' =  6.57  a      dist H1'-H8 =  3.80  a      dist_to_plane =  0.0756615        a
```

Figure S7. Output file of configurations. For each stem guanosine, the χ angle is given followed by the configuration based on its value, then the distances $d_{N3\text{-}O5'}$ and $d_{H1'\text{-}H8}$ followed by their respective predicted configuration, the distance of C1’ to the base plane represented by atoms (N9-C4-C8) and finally the retained configuration based on all these elements. This file corresponds to structure 2KPR. Note that dG12 has an undetermined configuration based on its χ angle and this indeterminacy is removed thanks to the distance calculations.

7D5E

Handedness

Left-handed helix

Topology

Block 1 consists of tetrads 1-2

Direction of the strands d d d d

Type Parallel

Block 2 consists of tetrads 3-4

Direction of the strands u u u u

Type Parallel

Bulges and Snapbacks

With a bulge between A4 and A6
With a bulge between A8 and A10
With a bulge between A13 and A15
With a 3'-bottom snapback of nt A26 in strand 4

Loops

Between strands 1-2	propeller loop bottom-top	(-plbt)	from A2	to A4	length 1 nt
Between strands 2-3	propeller loop bottom-top	(-plbt)	from A6	to A8	length 1 nt
Between strands 3-4	propeller loop bottom-top	(-plbt)	from A10	to A12	length 1 nt
Between strands 4-3	propeller loop top-bottom	(+pltb)	from A15	to A17	length 1 nt
Between strands 3-2	propeller loop top-bottom	(+pltb)	from A18	to A20	length 1 nt
Between strands 2-1	propeller loop top-bottom	(+pltb)	from A21	to A23	length 1 nt
Between strands 1-4	propeller loop top-bottom	(+pltb)	from A24	to A26	length 1 nt

Linkers between the two blocks

No linker in strand 1
No linker in strand 2
No linker in strand 3
Linker in strand 4 between A13 and A15

Number of tetrads = 4

Distribution of guanines in tetrads and strands

Strands		1	2	3	4
Distribution of guanines	tetrad 1	A 1	A 4	A 8	A 12
	tetrad 2	A 2	A 6	A 10	A 13
	tetrad 3	A 24	A 21	A 18	A 15
	tetrad 4	A 23	A 20	A 17	A 26
Glycosidic configuration	tetrad 1	a	a	a	a
	tetrad 2	a	a	a	a
	tetrad 3	a	a	a	a
	tetrad 4	a	a	a	a

Figure S8. Part of the results of ASC-G4 for 7D5E. In the Table the distribution of the stem guanines in the tetrads and strands is given, as well as the glycosidic configuration. In the columns, A refers to the chain name, followed by the guanine identification number. As observed, this 4-tetrad structure presents important discontinuities between tetrads 2 and 3 in strands 1, 2, and 3, which separate the two blocks. In the second block (tetrads 3 and 4), strand 4 consists of two nts: A15 and the snapback A26. Therefore, the direction of the strand is not obvious. However, since all gcs in this block are *anti* (without considering the snapback), indicating a parallel topology, and the direction of strands 1, 2, and 3 is up, it was deduced that the direction of strand 4 in block 2 is also up, resulting in a parallel topology. In the two blocks, the strands are connected by 7 loops. A linker, which is also a bulge between A13 and A15, connects the two blocks.

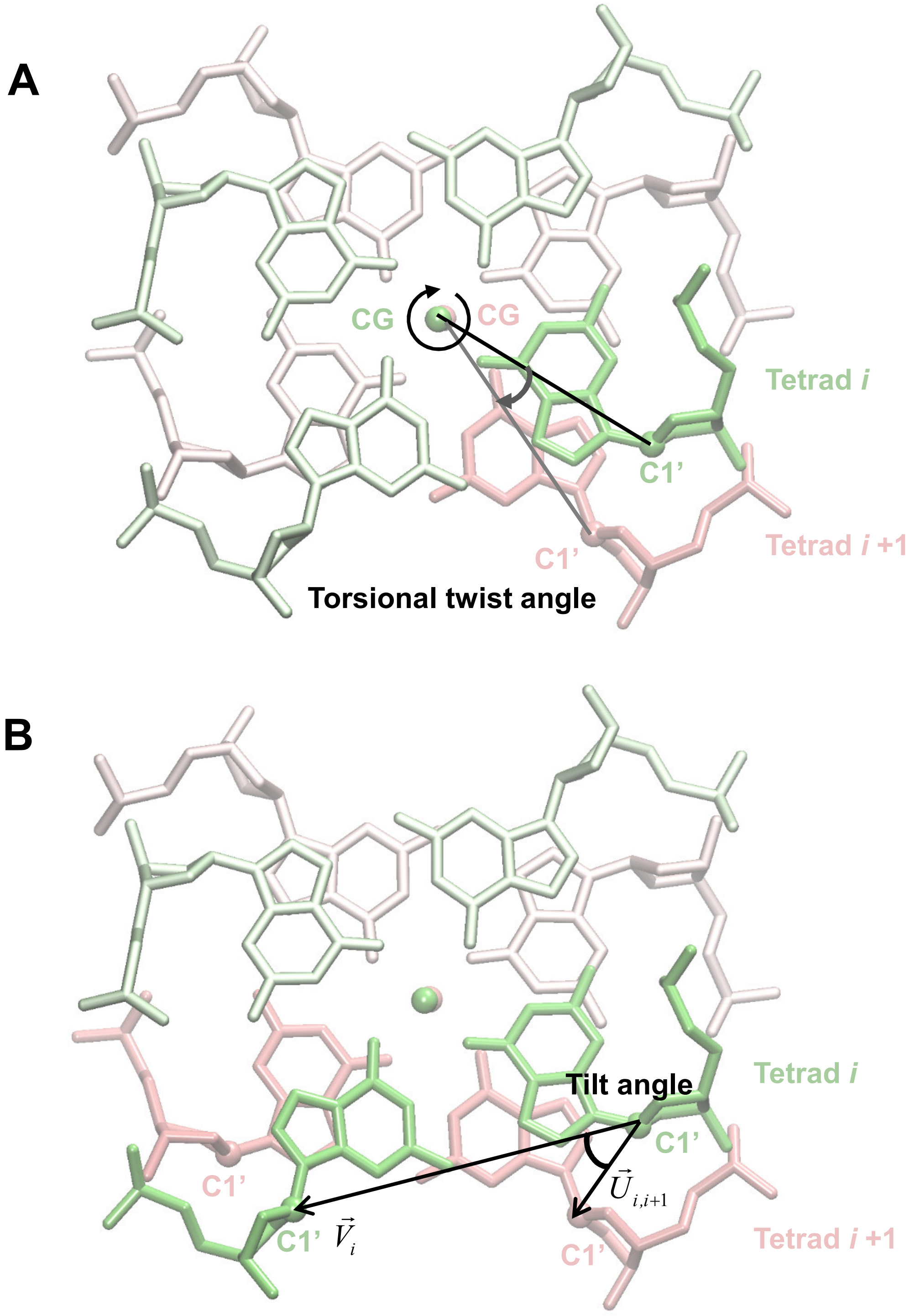

Figure S9. The torsional twist angle and the tilt angle. (A) The torsional twist angle is the pseudo-dihedral angle C1'(*i*)-CG(*i*)-CG(*i*+1)-C1'(*i*+1), where the first C1' and CG belong to tetrad *i* (in light green) and the second C1' and CG to tetrad *i*+1 (in pink). CG is the center of gravity of the four O6 atoms of a tetrad. The torsional twist angle is directional. (B) The tilt angle is the tetrad-strand angle calculated between vectors $\vec{V}_i$ and $\vec{U}_{i,i+1}$, the former corresponding to the tetrad and the latter to the strand. CG and C1' are shown as hard spheres.

```
groove 1  tetrad 1  min dist C3'-C3' =    15.636     for     A  11  -  A   7
groove 1  tetrad 2  min dist C3'-C3' =    12.415     for     A   1  -  A   7
groove 1  tetrad 3  min dist C3'-C3' =    11.037     for     A   2  -  A   7
groove 2  tetrad 1  min dist C3'-C3' =    14.814     for     A   7  -  A  18
groove 2  tetrad 2  min dist C3'-C3' =    10.933     for     A   6  -  A  18
groove 2  tetrad 3  min dist C3'-C3' =    11.112     for     A   5  -  A  18
groove 3  tetrad 1  min dist C3'-C3' =    13.912     for     A  18  -  A  14
groove 3  tetrad 2  min dist C3'-C3' =     9.996     for     A  17  -  A  14
groove 3  tetrad 3  min dist C3'-C3' =     8.672     for     A  16  -  A  14
groove 4  tetrad 1  min dist C3'-C3' =    11.845     for     A  14  -  A  11
groove 4  tetrad 2  min dist C3'-C3' =     7.143     for     A  13  -  A  11
groove 4  tetrad 3  min dist C3'-C3' =     6.105     for     A  12  -  A  11
---------------
groove 1  tetrad 1  min dist C5'-C5' =    17.668     for     A  11  -  A   7
groove 1  tetrad 2  min dist C5'-C5' =    14.456     for     A   1  -  A   7
groove 1  tetrad 3  min dist C5'-C5' =    11.781     for     A   2  -  A   7
groove 2  tetrad 1  min dist C5'-C5' =    15.619     for     A   7  -  A  18
groove 2  tetrad 2  min dist C5'-C5' =    12.200     for     A   6  -  A  18
groove 2  tetrad 3  min dist C5'-C5' =    12.634     for     A   5  -  A  18
groove 3  tetrad 1  min dist C5'-C5' =    15.247     for     A  18  -  A  14
groove 3  tetrad 2  min dist C5'-C5' =    11.361     for     A  17  -  A  14
groove 3  tetrad 3  min dist C5'-C5' =     8.193     for     A  16  -  A  14
groove 4  tetrad 1  min dist C5'-C5' =     9.528     for     A  14  -  A  11
groove 4  tetrad 2  min dist C5'-C5' =     6.985     for     A  13  -  A  11
groove 4  tetrad 3  min dist C5'-C5' =     6.352     for     A  12  -  A  11
```

Figure S10. Output file of the minimum groove widths. The groove widths are calculated from C3' atoms (upper part) and C5' atoms (lower part). For each groove and each tetrad, the minimum distance between guanine *i* of strand *n* (first guanine identification) and guanine *j* of strand *n*+1 (second guanine identification), is given. The interest of this file is not the minimum distance itself, which is also found in the final output file, but the nt identification of the closest guanines in each groove. This file corresponds to structure 2KPR.

Main chain torsion angles (definition)

Nucleotide	α	β	γ	δ	ε	ζ
A 1 DG	-	-	46.098	140.010	-171.991	-91.398
A 2 DG	-80.947	178.115	61.669	138.796	173.137	-104.360
A 3 DG	137.184	-153.127	-160.680	143.615	-177.914	-106.538
A 4 DT	-67.413	-154.827	56.086	145.890	-170.814	114.064
A 5 DG	70.280	-135.378	-178.351	149.098	173.998	-96.382
A 6 DG	-55.819	158.846	74.231	124.578	-178.887	-114.907
A 7 DG	-51.801	-174.851	48.362	143.766	-157.784	-95.489
A 8 DG	-68.560	163.964	61.398	145.201	-164.440	-142.116
A 9 DA	-55.274	142.135	68.328	141.052	-154.356	154.135
A 10 DA	68.140	-144.239	-171.485	153.763	-154.575	-151.073
A 11 DG	76.307	-110.507	-151.636	86.333	-111.446	-83.659
A 12 DG	-99.431	-128.854	54.696	100.223	-135.878	-46.366
A 13 DG	109.501	-167.864	-172.218	143.233	-175.003	-119.994
A 14 DG	-54.257	-169.719	49.217	142.465	-73.911	102.355
A 15 DT	62.341	-178.594	29.117	139.048	-126.582	159.115
A 16 DG	53.093	-136.310	35.181	139.958	-141.575	-79.408
A 17 DG	-98.378	152.949	90.267	129.797	-170.365	-125.003
A 18 DG	-109.613	173.686	74.168	85.866	-156.415	-53.511
A 19 DT	-85.155	-177.876	66.132	82.474	-	-

Sugar torsion angles (definition)

Nucleotide	ν0	ν1	ν2	ν3	ν4	ψ_{max}	P
A 1 DG	-27.667	38.334	-33.904	18.611	5.441	38.002	153.147
A 2 DG	-29.972	40.528	-35.139	18.597	6.778	39.979	151.516
A 3 DG	-28.172	40.733	-37.155	21.726	3.695	40.666	156.016
A 4 DT	-20.289	35.360	-36.224	25.195	-3.296	37.283	166.309
A 5 DG	-18.647	35.187	-37.316	27.290	-5.645	37.929	169.687
A 6 DG	-36.734	38.486	-26.030	5.610	19.196	38.860	132.054
A 7 DG	-19.725	33.337	-33.611	22.828	-2.123	34.830	164.798
A 8 DG	-25.384	39.313	-37.690	23.783	0.747	40.047	160.244
A 9 DA	-25.601	37.311	-34.366	20.318	3.091	37.434	156.639
A 10 DA	-11.417	30.870	-37.555	31.626	-12.810	37.561	181.065
A 11 DG	-0.095	-22.567	35.184	-35.843	22.725	37.087	18.436
A 12 DG	-32.953	17.690	2.346	-21.141	34.163	34.504	86.102
A 13 DG	-31.648	44.121	-38.968	21.503	5.895	43.596	153.360
A 14 DG	-22.771	35.473	-34.236	21.875	0.416	36.262	160.755
A 15 DT	-31.078	41.541	-35.595	18.178	7.960	40.964	150.334
A 16 DG	-29.664	40.321	-35.160	18.503	6.890	39.950	151.656
A 17 DG	-38.203	42.663	-30.717	9.822	17.649	42.119	136.827
A 18 DG	-7.372	-15.354	30.611	-35.269	26.936	35.319	29.923
A 19 DT	-13.647	-11.230	29.982	-38.770	33.090	38.488	38.833

Figure S11. Output of the main chain and sugar torsion angles. This file corresponds to structure 2KPR.

Table S1. Distribution of the topologies of the 192 one-block and interlaced structures.

Topology	Number of structures	PDB ID
Parallel	85	1KF1, 1MY9[D], 1MYQ[D], 1XAV, 1Y8D[I], 2A5P[3'b], 2A5R[3'b], 2KQG, 2KQH, 2KYP, 2KZE, 2L7V, 2L88, 2LBY, 2LD8, 2LE6[D], 2LEE, 2LK7, 2LPW, 2LXQ, 2LXV[D], 2M27, 2M27, 2M4P, 2M53, 2M90[5't], 2M92[3'b], 2M93, 2MB2, 2MB4[D,5'b], 2MGN[3'b], 2N21, 2N4Y, 2N60, 2N6C[3'b], 2O3M[3'b], 2RQJ[D], 2RSK[D], 2RU7[D], 3CDM, 3QXR[3'b], 3R6R, 3SC8, 3T5E, 3UYH, 4DA3, 4DAQ, 4FXM, 4G0F, 4WO2[D,3'b], 4WO3, 5BJO, 5BJP, 5CCW, 5DWW[D], 5DWX, 5I2V, 5LIG, 5NYS, 5NYT, 5NYU, 5UA3[D], 5VHE, 5W77, 6AU4, 6E80, 6E81, 6E84, 6ERL, 6FQ2[D,L,3't,5'b], 6H5R, 6IP3, 6IP7, 6ISW, 6JJ0, 6JWE, 6K3Y, 6NEB, 6O2L, 6SUU[3'b], 6T2G, 6T51, 7CLS, 7KBV, 7LL0
Antiparallel-chair	37	148D, 1BUB, 1C32, 1C34, 1C35, 1C38, 1HAO, 1HAP, 1HUT, 1QDF, 1QDH, 1RDE, 2IDN, 2KM3, 2LYG, 2M8Z, 2N2D, 3QLP, 4DIH, 4DII, 4LZ1, 4LZ4, 4NI7, 4NI9, 5CMX, 5EW1, 5EW2, 5MJX, 5OPH, 5YEY, 6EO6, 6EO7, 6EVV, 6FC9, 6GH0, 6GN7, 6JKN
Antiparallel-basket	19	143D, 1I34, 201D, 230D, 2KF7, 2KF8, 2KKA, 2M6V, 2M6W, 2M91, 2MCC[RL], 2MCO, 2MFT, 5J05, 5J4P, 5J4W, 5J6U, 5LQG, 6GZN
Antiparallel-basket2	12	2KOW, 2MBJ, 4KZD, 4KZE, 4Q9Q, 4Q9R, 5LQH, 5OB3, 6B14, 6B3K, 6F4Z, 6FTU
Hybrid1	12	2E4I, 2GKU, 2HY9, 2JSK, 2JSM, 2MAY, 2MB3, 2MWZ, 5MBR, 5Z80, 6IA4, 6XT7[5't]
Hybrid2	11	2LOD, 5MCR, 5MTA, 5MTG, 5OV2, 6E8S, 6E8T, 6E8U, 6FFR, 6JCD[3't], 6L92
Hybrid3	11	186D, 2F8U, 2JPZ, 2JSL, 2JSQ, 2KZD, 2MFU, 5MVB, 6AC7, 6CCW, 6IA0
Hybrid4	5	2KPR[5'b], 5O4D[5'b], 6H1K[5'b], 6L8M[5'b], 6R9L

[L] Left-handed helix
[RL] Hybrid Right-handed / Left-handed helix
[D] Stacking-stem dimer
[I] Interlaced dimer
[3'b] Structure with a 3'-bottom snapback
[3't] Structure with a 3'-top snapback
[5'b] Structure with a 5'-bottom snapback
[5't] Structure with a 5'-top snapback

Table S2. Distribution of the topologies of the 15 two-block structures. The topology and the direction of the strands correspond to Block 1 / Block2.

Topology	Number of structures	PDB ID
Parallel / Parallel<br>dddd / dddd	1	2N3M
Parallel / Parallel<br>dddd / uuuu	4	2MS9[L,3'b], 4U5M[L,3'b], 6GZ6[L,3'b,5'b],<br>7D5E[L,3'b]
Parallel / Parallel<br>uuuu / dddd	1	1OZ8[RL]
Parallel / Hybrid2<br>uuuu / dddu	1	6KVB[3't,5'b]
Parallel / −<br>dddd / one tetrad	4	5V3F[D], 6C63[D], 6C64[D], 6C65[D]
− / Parallel<br>one tetrad / uuuu	4	2LA5[RL], 5DE5[RL], 5DE8[RL], 5DEA[RL]

[L] Left-handed helix
[RL] Hybrid Right-handed / Left-handed helix
[D] Stacking-stem dimer
[I] Interlaced dimer
[3'b] Structure with a 3'-bottom snapback
[3't] Structure with a 3'-top snapback
[5'b] Structure with a 5'-bottom snapback
[5't] Structure with a 5'-top snapback

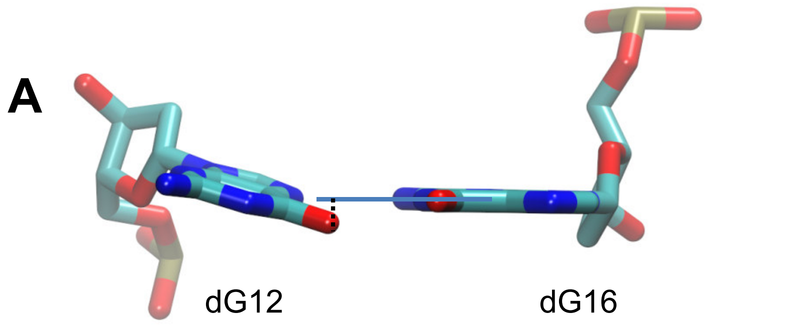

6CCW

Distance of dG12:O6 to dG16:plane (N1-O6-N7) = 1.0 Å

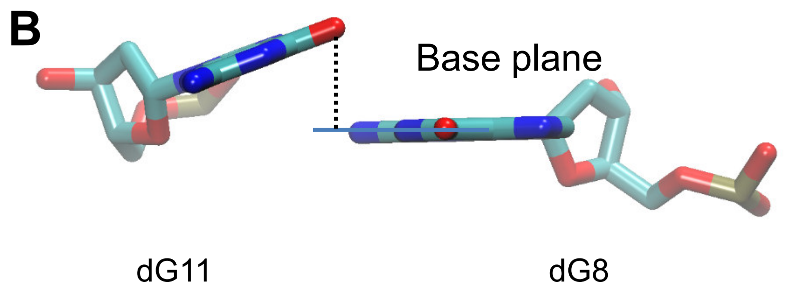

1OZ8

Distance of dG11:O6 to dG8:plane (N1-O6-N7) = 2.8 Å

Figure S12. The two extreme non-coplanar Hoogsteen base-pairs of guanines. (A) 6CCW has the smallest distance between atom O6 and the facing base plane in our range of visually detectable non-coplanar pairs. (B) 1OZ8 has the largest distance O6 to the facing base plane in our set of G4 structures. The atoms' color code: C (cyan), N (blue), O (red), and P (tan). The blue plain line represents the base plane and the black dashed line the distance of O6 to this plane. Hydrogen atoms are omitted for clarity.

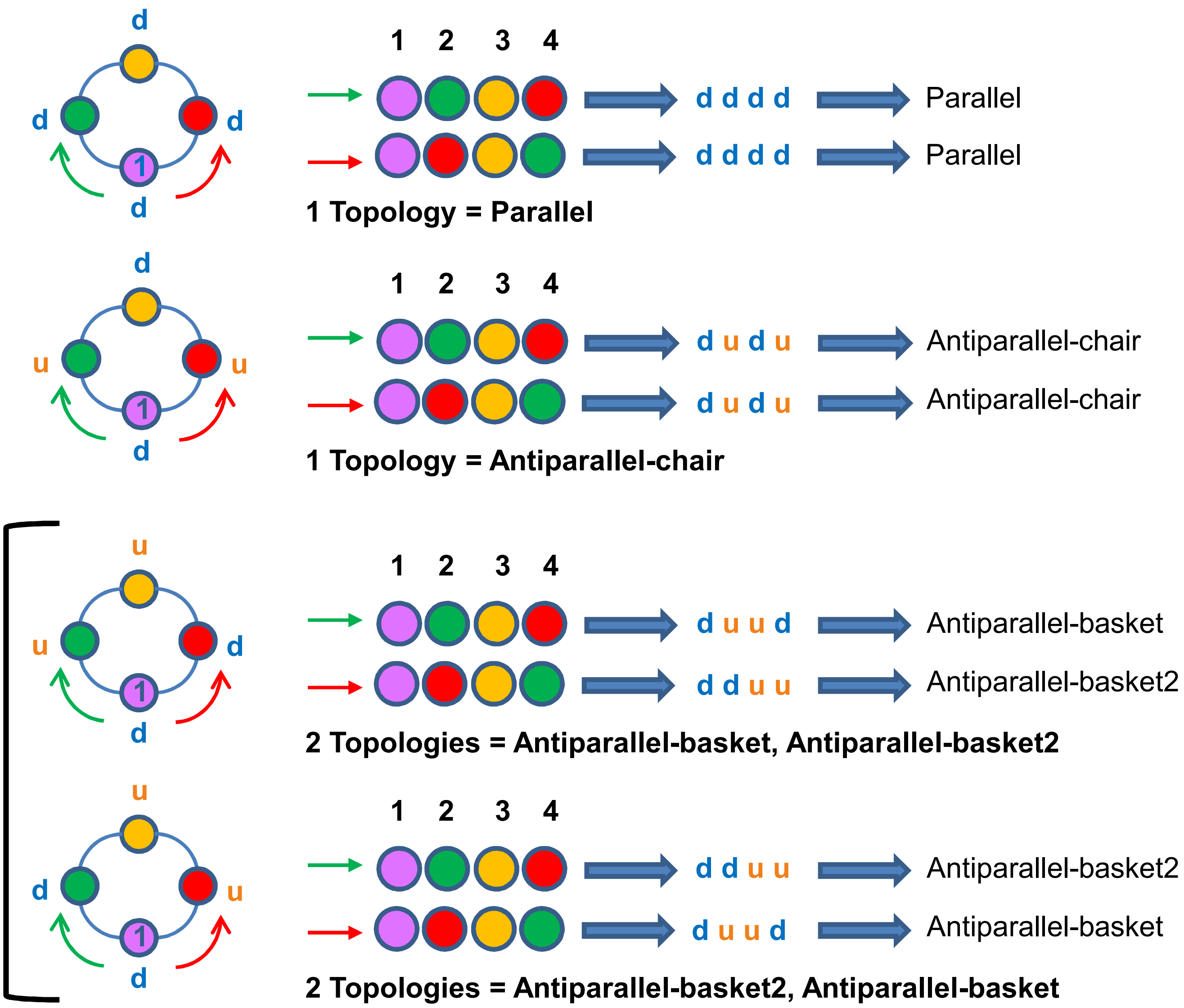

Figure S13 (part1). Investigation of the difference in the topologies with both clockwise (green arrows) and anticlockwise (red arrows) orientations of the strand numbering. This is a schematic representation of G4 viewed from the top of the tetrad that contains the first stem-guanine. Each circle represents a strand. The first strand (in purple), which is always down, is indicated with the number 1. The strands are colored according to their position in the stem. The direction of the strands (down, **d**, and up, **u**) are indicated near them. On the right of each G4, the strands are dispatched linearly according to the direction of their **numbering** (clockwise, green arrow, and anticlockwise, red arrow). The number attributed to each strand is written above the dispatched strands. This is followed by the direction of the strands (**d** and **u**) and the resulting topology. As observed, in four cases, numbering the strands in the clockwise or the anticlockwise orientation yields to an unambiguous topology, parallel, antiparallel-chair, hybrid1, and hybrid4 (see next page for the hybrid topologies). Whereas in four other cases, numbering in one orientation or the other yields two different topologies that are currently confused, antiparallel-basket with antiparallel-basket2 and hybrid2 with hybrid3. These topologies are grouped using black square brackets. Please note that here, the clockwise and anticlockwise orientations are only used to help understand the drawing, whereas, in ASC-G4, the orientation of the strand numbering is based on the configuration of the first stem-guanine: it follows the H-bond donors for an *anti*-G and the H-bond acceptors for a *syn*-G. For most G4s this orientation corresponds to a clockwise direction.

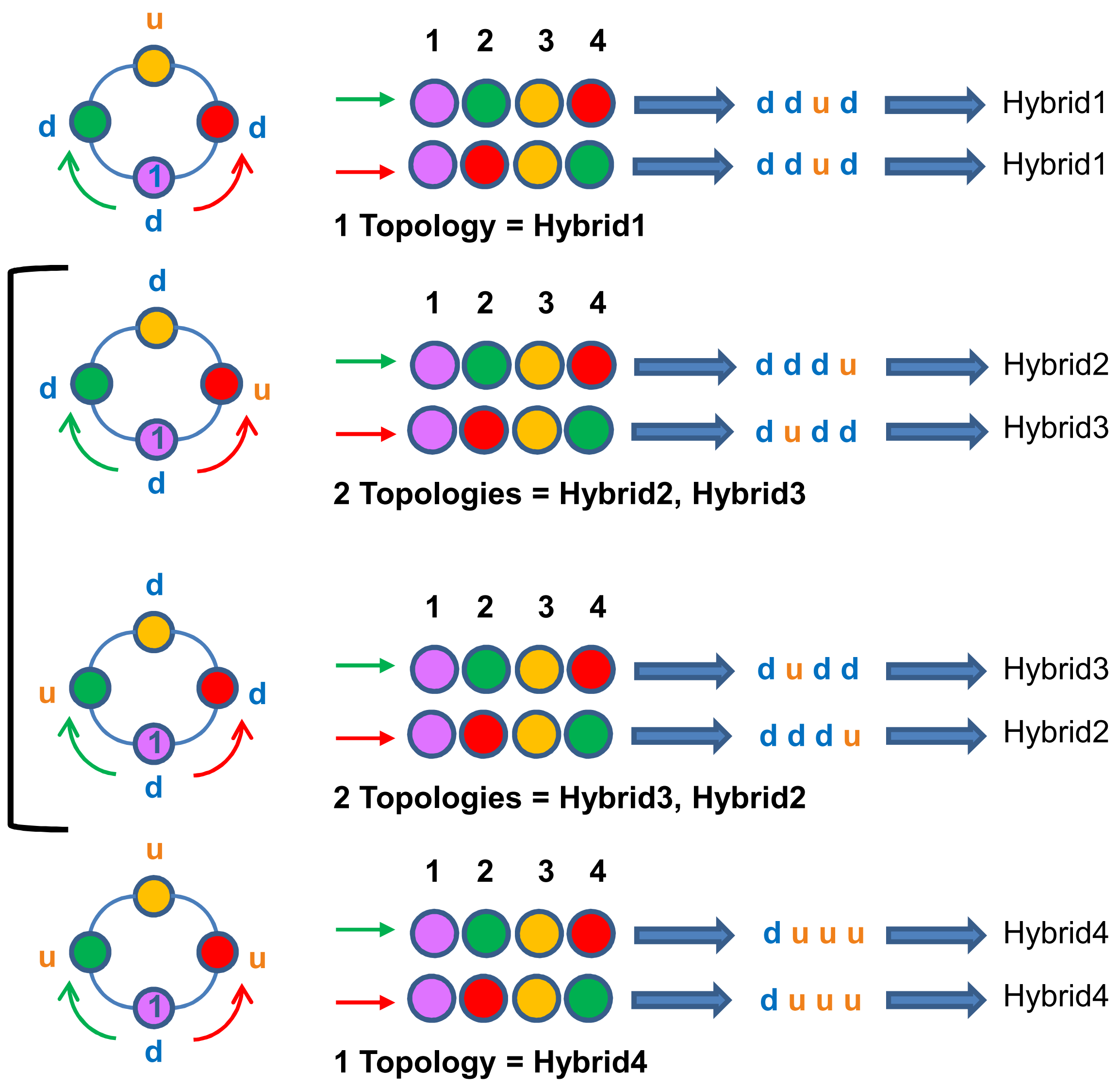

Figure S13 (part 2). Same as S13 (part 1) but for hybrid topologies.

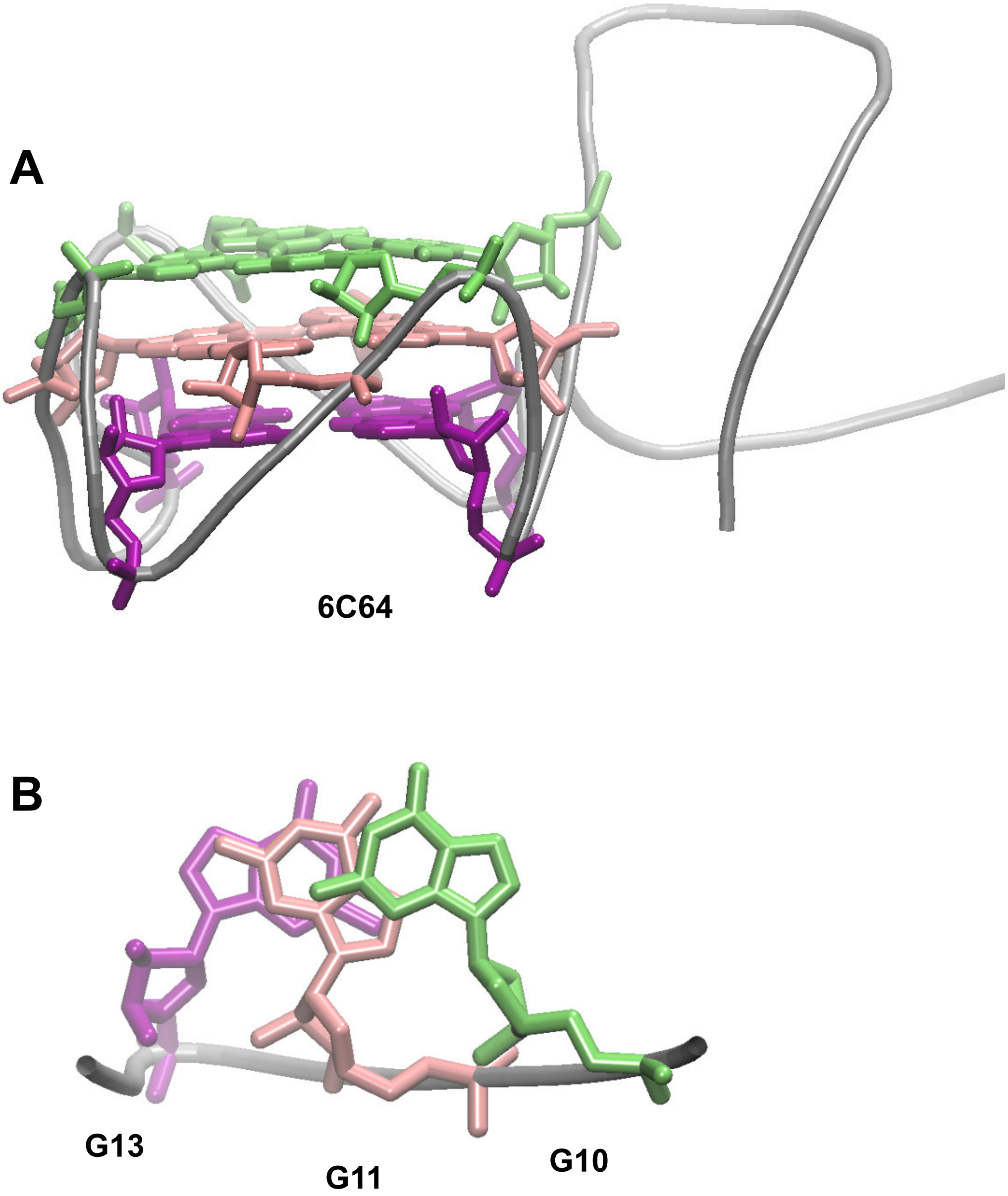

6C64

Figure S14. Representative structure of a two-block parallel/− topology, 6C64. (A) Lateral view of the structure. (B) Top view of the first strand, showing that although the three guanines are *anti*, they are nevertheless, with reversed orientations. Indeed, whereas the bases of G10 and G11 are oriented as usual to the left, the base of G13 is oriented to the right. In (A) and (B) the backbone is depicted as a gray tube, tetrad 1 is in light green, tetrad 2 in pink, and tetrad 3 in purple. Hydrogen atoms are omitted for clarity.

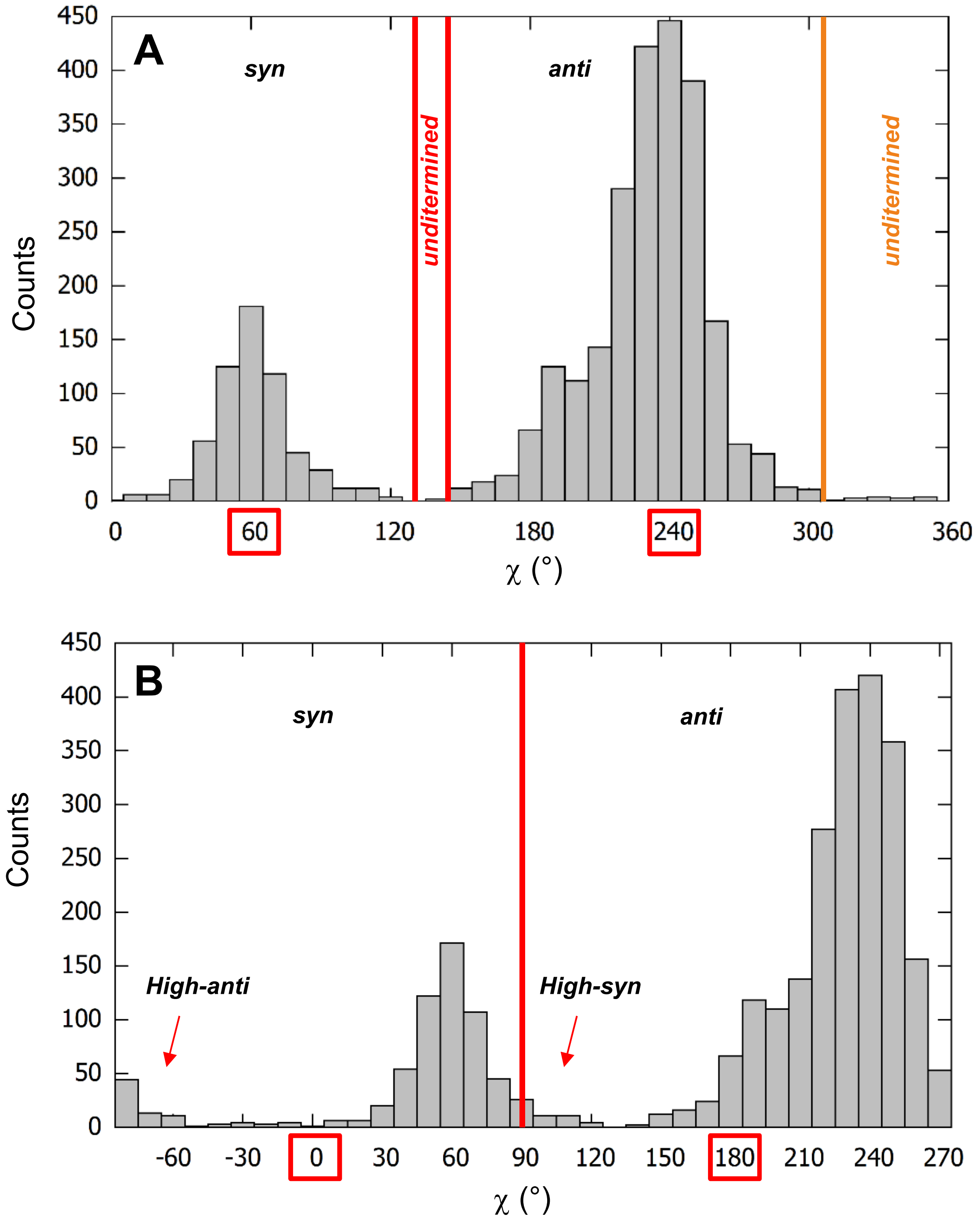

Figure S15. Histogram of GBA depicted according to two different ranges. (A) The same histogram as in Figure 4 of the main text is presented here for comparison. The peaks of the bimodal distribution are surrounded by red boxes. (B) The same histogram as in (A) shifted to coincide with the IUPAC-IUB ranges of *syn*-G and *anti*-G. The *high-syn* and *high-anti* intervals are indicated with arrows. They correspond to the tails of the unimodal distributions. The center of each range is surrounded by a red box. In (A) and (B), the intervals are delimited by red and orange vertical lines.

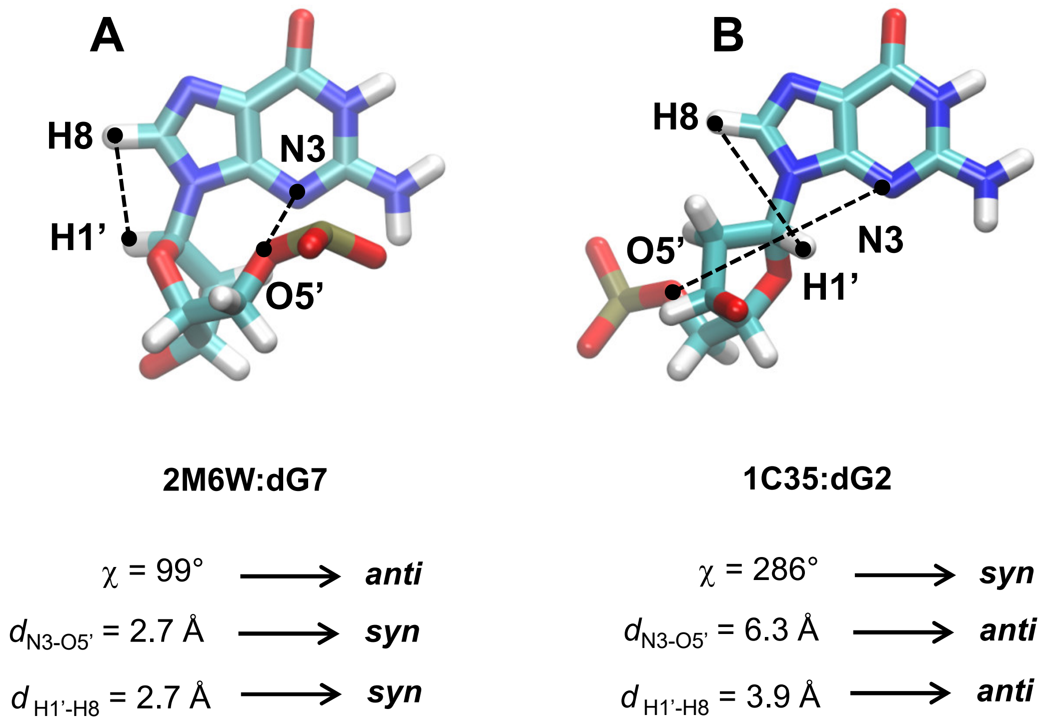

Figure S16. Illustration of the discrepancies between the different characteristics of the configuration. Two guanosines to illustrate the discrepancy between the configuration given by the χ angle, when following the IUPAC-IUB definition, i.e., *syn* for 0 ± 90° (]270°,90°]) and *anti* for 180 ± 90° (]90°,270°]), and the distances between atoms N3 and O5’, and H1’ and H8. These discrepancies are removed when the χ angle is in the ranges we finally adopted, i.e., *syn* for]0°,140°] and *anti* for]152°,300°]. The atoms' color code is C (cyan), N (blue), O (red), P (tan), and H (white).
